# Broad-Spectrum Antiviral Activity of Cyclophilin Inhibitors Against Coronaviruses: A Systematic Review

**DOI:** 10.3390/ijms26167900

**Published:** 2025-08-15

**Authors:** Abdelazeem Elhabyan, Muhammad Usman S. Khan, Aliaa Elhabyan, Rawan Abukhatwa, Hadia Uzair, Claudia Jimenez, Asmaa Elhabyan, Yee Lok Chan, Basma Shabana

**Affiliations:** 1 Institute for Regeneration and Repair, University of Edinburgh, Edinburgh EH16 4UU, UK; 2 Faculty of Medicine, Tanta University, Tanta 31111, Egypt; 3 Department of Pathology, Faculty of Medicine, Tanta University, Tanta 31111, Egypt; 4 Faculty of Veterinary Science, University of Veterinary and Animal Sciences, Lahore 54000, Pakistan; 5 Deanery of Biomedical Sciences, The University of Edinburgh, Edinburgh EH8 9YL, UK; 6 Dermatology Department, Faculty of Medicine, Menoufia University, Shebeen El-Kom 6132720, Egypt

**Keywords:** cyclophilin inhibitors, cyclosporine A, alisporivir, broad-spectrum anti-viral, coronaviruses, SARS-CoV, MERS-CoV, SARS-CoV-2, HCoV-229E, viral replication

## Abstract

Cyclophilins (Cyps), a family of peptidyl-prolyl isomerases, play essential roles in the life cycle of coronaviruses by interacting with viral proteins and modulating host immune responses. In this systematic review, we examined cell culture, animal model, and clinical studies assessing the anti-viral efficacy of cyclosporine A (CsA, PubChem CID: 5284373) and its non-immunosuppressive derivatives against coronaviruses. CsA demonstrated robust anti-viral activity in vitro across a broad range of coronaviruses, including but not limited to HCoV-229E, SARS-CoV, MERS-CoV, and SARS-CoV-2, with potent EC_50_ values in the low micromolar range. Non-immunosuppressive analogs such as Alisporivir and NIM811 exhibited similar inhibitory effects. In vivo, CsA treatment significantly reduced viral load, ameliorated lung pathology, and improved survival in coronavirus-infected animals. Clinical studies further indicated that CsA administration was associated with improved outcomes in COVID-19 patients, including reduced mortality and shorter hospital stays. Mechanistic studies revealed that CsA disrupts the formation of viral replication complexes, interferes with critical Cyp–viral protein interactions, and modulates innate immune signaling. These findings collectively demonstrate the therapeutic potential of cyclophilin inhibitors as broad-spectrum anti-virals against current and emerging coronaviruses.

## 1. Introduction

Viral infections remain a significant global health challenge, causing a spectrum of diseases that can range from mild to life-threatening [1,2]. The continuous emergence of viral pathogens, such as the coronaviruses responsible for SARS, MERS, and the recent COVID-19 pandemic, underscores the urgent need for effective anti-viral therapies [3]. These outbreaks have not only strained healthcare systems worldwide [4] but also highlight the critical importance of advancing our understanding of anti-viral agents and their mechanisms of action [5]. Cyclosporines, traditionally used as immunosuppressive agents in organ transplantation, have garnered attention for their potential anti-viral properties. Studies have suggested that cyclosporines may inhibit the replication of various viruses, including coronaviruses, by targeting essential viral and host factors involved in the replication process [6]. Cyclophilins, a family of peptidyl-prolyl isomerases, are among these crucial host factors [7]. They play crucial roles in protein folding and trafficking, and also contribute to various cell signaling pathways, impacting processes such as cell proliferation, migration, differentiation, and immune signaling [8]; cyclophilins such as cyclophilin D (CypD) are key regulators of the mitochondrial permeability transition pore (MPTP), and their activity significantly influences cell death pathways, particularly in the context of disease [9]. These processes are often hijacked by viruses to facilitate their replication. Given the pivotal role of cyclophilins in viral replication, they represent promising targets for anti-viral interventions [10,11]. Despite the promising anti-viral potential of cyclosporines—which target pro-viral cyclophilins—there is a lack of comprehensive reviews that systematically evaluate their impact on viral replication. Addressing this gap is essential to inform future research and therapeutic development. Understanding how these agents modulate viral replication could pave the way for novel anti-viral strategies, particularly in the context of coronavirus infections, where effective treatments are still urgently needed. This systematic review aims to synthesize experimental and clinical evidence on the impact of cyclosporines and cyclophilins on viral replication. Specifically, we will evaluate their effects on viral replication in cell culture, animal models, and clinical studies, and provide mechanistic insights into their modes of action. By collating and analyzing the available evidence, this review seeks to contribute to the development of targeted anti-viral therapies that can enhance our preparedness and response to viral outbreaks.

## 2. Methods

**Figure 1:**
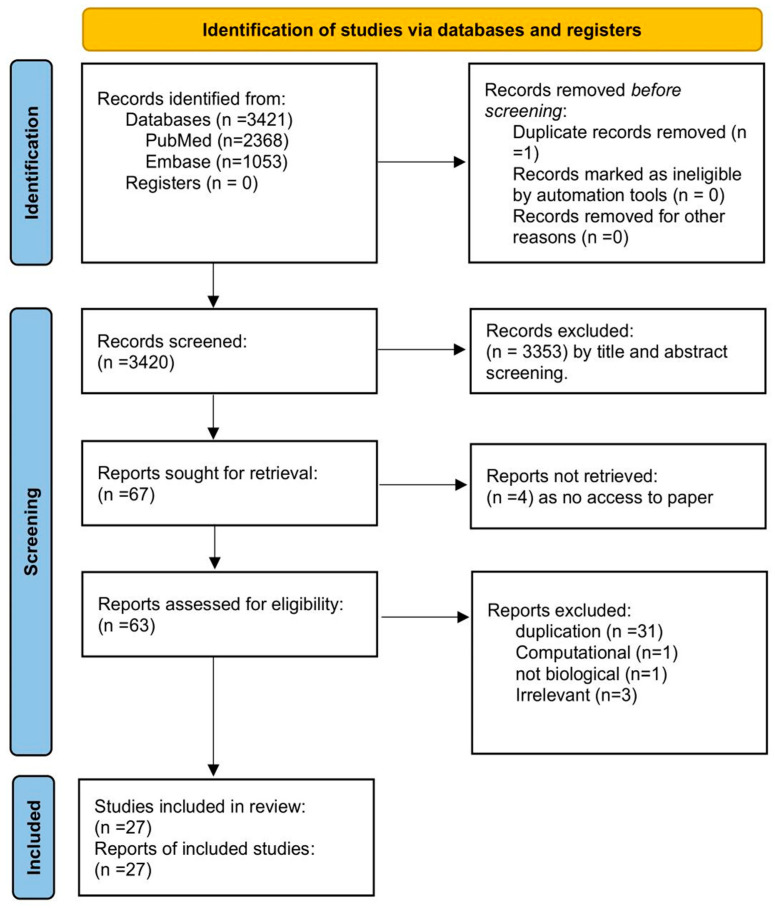
Flow diagram of the study selection process. The database search yielded 2368 records from PubMed and 1053 records from Embase, resulting in a total of 3420 records after duplicate removal. Title screening was conducted for 3420 studies, of which 808 were selected for abstract screening. All 63 abstracts met the inclusion criteria and were further assessed via full-text review. Ultimately, 27 full-text articles fulfilled the eligibility criteria and were included in the systematic review, each represented by a single report. Therefore, the number of reports included was also 27.

This systematic review was conducted and reported in accordance with the Preferred Reporting Items for Systematic Reviews and Meta-Analyses (PRISMA) guidelines. The study selection process is illustrated in the PRISMA flow diagram (Figure 1). The review protocol was not registered.

### 2.1. Search Strategy: (As Shown in Table 1)

A comprehensive literature search was conducted to identify studies investigating the impact of cyclosporines on viral replication and the modulation of viral replication by cyclophilins, as shown in Figure 1. The search was carried out across two major databases: PubMed and Embase, up to [15 March 2024]. The search strategy was designed to include a broad range of terms related to viruses, viral replication, cyclophilins, and cyclosporines, with adjustments made for each database’s indexing system and search capabilities.

PubMed Search: The following MeSH terms and field tags were utilized, yielding 2368 results.

EMBASE Search: Employed a combination of keywords, resulting in 1053 results.

**Table 1:** Database search strategies and results.

Database	Search Strategy	Results
PubMed	(“Viruses” [Mesh] OR “Virus Diseases” [Mesh] OR “Virus Replication” [Mesh] OR “Virology” [Mesh] OR “Virus Assembly” [All Fields] OR “Viral Assembly” [All Fields]) AND (“Cyclophilins” [MeSH Terms] OR “Peptidylprolyl Isomerase” [Mesh] OR “Cyclosporine” [Mesh] OR “Cyclosporins” [Mesh]) NOT “Review” [Publication Type]	2368
Embase	(‘viruses’ OR ‘virus diseases’ OR ‘virus replication’ OR ‘virology’ OR ‘virus assembly’ OR ‘viral assembly’) AND (‘cyclophilins’ OR ‘peptidylprolyl isomerase’ OR ‘cyclosporine’ OR ‘cyclosporins’)	1053

### 2.2. Study Selection

#### 2.2.1. Duplication Check

Using Excel’s conditional formatting feature, reviewers highlighted and identified duplicates within and across both databases to ensure the review’s integrity. No duplicates were identified between PubMed and EMBASE databases, except for a single duplicate within the EMBASE results.

#### 2.2.2. Screening Titles

Two reviewers independently screened titles from PubMed and EMBASE databases. This step aimed to identify potentially relevant papers discussing the effects of cyclosporines on viral replication and the modulation of viral replication by cyclophilins, in line with the review’s objectives. A total of 808 titles were found to be aligned with our focus on the effect of cyclophilins and cyclophilin inhibitors on coronavirus replication.

#### 2.2.3. Screening Abstracts

Following the title screening and duplication check, abstracts of the selected papers were reviewed by the two reviewers independently. Predefined inclusion criteria aimed at identifying studies directly addressing the review topics were applied to narrow down the selection. Out of the 808 screened titles, only 67 studies matched our inclusion criteria.

### 2.3. Inclusion Criteria

Study Population: Studies investigating the effect of cyclophilins and cyclophilin inhibitors on coronavirus replication.

#### Types of Studies

Tissue Culture Studies focusing on the effects of cyclosporine and other cyclophilin inhibitors on coronavirus replication.

Animal Model Studies assessing the impact of cyclosporines and other cyclophilin inhibitors on coronavirus replication.

Clinical Studies evaluating the effects of cyclosporines and other cyclophilin inhibitors on viral infections in humans.

Interventions: Studies involving the administration of cyclosporine and/or other cyclophilin inhibitors.

Outcomes: Primary outcomes include measures of viral replication, infection rates, and clinical outcomes in humans, among others.

Language and Publication Date: Studies published in English, with no restriction on publication date.

### 2.4. Exclusion Criteria

#### 2.4.1. Types of Publications

Review articles, systematic reviews, and meta-analyses studies.

#### 2.4.2. Study Focus

Studies focusing on the effects of cyclosporines on non-viral infections (e.g., tuberculosis, bacterial infections).

Research involving interventions other than cyclosporine and cyclophilin inhibitors, such as IVIG (Intravenous Immunoglobulin) and direct anti-virals.

Studies examining the effects of Tacrolimus on viral infections are excluded to maintain a focused review on cyclosporine and cyclophilin inhibitors.

### 2.5. In-Depth Analysis

The 27 research papers included in our systematic review underwent an extensive review. This step included a full-text examination to gather relevant data, conclusions, and insights regarding the roles and mechanisms of cyclosporines and cyclophilins in viral replication.

### 2.6. Data Extraction

Data from included studies were extracted by two independent reviewers and peer-reviewed for accuracy. The extraction focused on study characteristics, methodologies, results, and conclusions pertinent to the effects of cyclosporines and cyclophilins on viral replication. Any discrepancies in data extraction and interpretation were resolved through discussion or consultation with a third reviewer.

The database search yielded 2368 records from PubMed and 1053 records from Embase, resulting in a total of 3420 records after duplicate removal. Title screening was conducted for 3420 studies, of which 808 were selected for abstract screening. All 67 abstracts met the inclusion criteria and were further assessed via full-text review. Ultimately, 27 full-text articles fulfilled the eligibility criteria and were included in the systematic review. A flow diagram of the study selection process is shown in Figure 1.

Given the biological and mechanistic nature of the included studies, formal risk of bias assessment tools and quantitative effect measures commonly used in clinical systematic reviews were not applicable. Data synthesis was performed narratively, supported by tabulated summaries and visual presentations. No meta-analysis was conducted due to heterogeneity in study designs and outcomes.

## 3. Results and Discussion

Antiviral therapies often target viral proteins like reverse transcriptases and proteases. However, their efficacy can be compromised by the high mutation rates of viruses, particularly RNA viruses, which rapidly develop resistance. Since many viruses rely on common host cell factors and pathways for replication, targeting these shared host mechanisms can provide activity against multiple viral pathogens [12]. This is especially valuable for emerging viruses or outbreaks where specific anti-virals may not be available. Since host factors are evolutionarily conserved, viruses would need to undergo substantial mutations to evade HDTs, which is less likely compared to direct-acting anti-virals [12]. By disrupting key cellular processes involved in viral entry and replication, host-targeting drugs offer a means to impair viral infection with a reduced risk of resistance development, as they focus on host factors rather than viral components [13]. Cyclophilins are a family of intracellular enzymes critical for the correct folding and functionality of several proteins, impacting both cellular and viral proteins [14]. Several classes of immunosuppressive and non-immunosuppressive cyclophilin inhibitors have shown promising results in the inhibition of viral replication in vivo and in vitro [15]. The effectiveness of (CsA) and non-immunosuppressive cyclosporines in inhibiting coronavirus replication has been validated through extensive research. As summarized in (Supplementary Table S1 and Table 2, Table 3 and Table 4), these compounds exhibit robust anti-viral activity across tissue culture systems, animal models, and clinical trials. Moreover, the involvement of cyclophilins in coronavirus replication, as illustrated in (Supplementary Table S2), highlights the mechanistic insights gained from current studies, reinforcing the potential of these targets in therapeutic development.

### 3.1. Broad-Spectrum Antiviral Activity of Cyclophilin Inhibitors on Different Strains of Coronavirus (Supplementary Table S1, Table 2 and Table 3)

Cyclophilin inhibitors, particularly (CsA), Alisporivir (ALV), and NIM811, have demonstrated substantial broad-spectrum anti-viral efficacy against various coronavirus strains. These compounds effectively suppress viral replication at micromolar concentrations across different cell lines and experimental models, highlighting their potential as therapeutic agents. Studies have consistently shown that CsA, ALV, and NIM811 potently inhibit the replication of multiple coronaviruses, including HCoV-229E, SARS-CoV, SARS-CoV-2, and MERS-CoV [16,17,18,19]. For instance, ALV achieved viral reduction by up to 5 logs in Vero cells infected with various SARS-CoV strains, with EC50 values ranging from 1.3 to 8.3 µM [20]. Similarly, CsA demonstrated significant inhibition of SARS-CoV-2 replication in human bronchial epithelial cells and precision-cut lung slices at concentrations around 10 µM [19]. The broad-spectrum activity of these inhibitors extends to different coronavirus genera and is maintained across various cell types, including primary human cells. This consistency suggests that cyclophilin inhibitors target a conserved mechanism in coronavirus biology, likely related to their modulation of cyclophilins, a cellular protein essential for viral replication [16]. Importantly, ALV has shown a more favorable cytotoxicity profile compared to CsA, particularly in studies with SARS-CoV-1 and SARS-CoV-2 [17]. A summary of reported half-maximal effective concentrations (EC50) and half-maximal cytotoxic concentrations (CC50) from in vitro studies is provided in Table 2. This table consolidates data previously spread across Supplementary Table S1, facilitating direct comparison across compounds and viral strains. Not all studies reported CC50 or TI values, which limit full cross-study comparisons; future research should aim to systematically assess these parameters for consistent benchmarking of anti-viral potency and safety. This improved safety profile, combined with its potent anti-viral activity, positions ALV as a promising candidate for further clinical development. The efficacy of these compounds across multiple coronavirus strains, including newly emerged variants, underscores their potential as broad-spectrum anti-virals. Their ability to inhibit viral replication at various stages of the infection cycle suggests they could be valuable tools in combating current and future coronavirus outbreaks. Furthermore, the potential for combination therapy with other anti-viral agents opens up additional avenues for therapeutic intervention [17,19]. Combination therapies involving cyclophilin inhibitors have shown promising results. The synergistic effect of cyclosporine (9 µM) and IFN alfacon-1 (2.4 × 10^4^ U/mL) significantly reduced MERS-CoV replication in Vero E6 cells, with ex vivo studies in bronchus and lung tissues corroborating these findings [2]. Similarly, the combination of interferon-alpha1 (IFN-α1) and (CsA) potently inhibited MERS-CoV (EMC) replication in human microvascular endothelial cells (HMVEC-L) [2]. These results suggest that combining cyclophilin inhibitors with interferons may enhance their anti-viral efficacy. The differential sensitivity of coronaviruses to interferons is noteworthy. MERS-CoV demonstrated a 50–100-fold increased response to PEG-IFN treatment compared to SARS-CoV in Huh7 cells [21]. This heightened sensitivity of MERS-CoV to interferon treatment, coupled with the synergistic effects observed with cyclophilin inhibitors, presents a compelling rationale for further investigation of combination therapies.

**Table 2:** Summary of reported EC_50_ and CC_50_ values from in vitro studies evaluating cyclophilin inhibitors against coronaviruses. This table compiles EC_50_ (half-maximal effective concentration) and CC_50_ (half-maximal cytotoxic concentration) values reported for various cyclophilin inhibitors tested against different coronavirus strains in multiple cell lines. This table provides a summary of Supplementary Table S1. Additional studies listed in Supplementary Table S1 are not included here as they do not report EC_50_ and CC_50_ values.

Virus (Strain)	Cell Line	Compound	EC50 (µM)	CC50 (µM)	Reference
SARS-CoV-2	Vero E6	Alisporivir	0.46	-	[22]
MERS-CoV (EMC/2012)	Vero	Alisporivir	3.6 ± 1.1 ^1^/3.9 ± 1.7 ^2^	26.4	[20]
MERS-CoV (EMC/2012)	Huh7	Alisporivir	3.4 ± 1.0 ^1^/2.8 ± 1.0 ^2^	43.8	[20]
MERS-CoV (EMC/2012)	LLC-MK2	Alisporivir	4.0 ± 1.1 ^2^	14.3 ± 1.8	[20]
MERS-CoV (N3/Jordan)	Vero	Alisporivir	3.0 ± 1.0 ^1^	26.4 ± 1.0	[20]
MERS-CoV (N3/Jordan)	Huh7	Alisporivir	1.5 ± 1.0 ^1^	43.8	[20]
SARS-CoV (Frankfurt-1)	VeroE6	Alisporivir	8.3 ± 1.0 ^1^	>50	[20]
SARS-CoV (Frankfurt-1)	VeroE6	CsA	3.3	-	[23]
SARS-CoV (MA-15)	VeroE6	Alisporivir	1.3 ± 0.05 ^1^	>50	[20]
HCoV-NL63 WT	Caco-2	CsA	0.9–2.0	-	[24]
HCoV-NL63 WT	Caco-2	Alisporivir	0.8	-	[24]
HCoV-NL63 WT	Caco-2	NIM811	0.8	-	[24]
HCoV-NL63 WT	Caco-2	Compound 3	1.1	-	[24]
HCoV-NL63 WT	Caco-2	FK508 (Tacrolimus)	6.6	-	[24]
HCoV-229E-Luc	HuH-7.5	CsA	2.09/0.97 ^3^	-	[16]
HCoV-229E-Luc	HuH-7.5	Alisporivir	2.77/1.37 ^3^	-	[16]
HCoV-229E-Luc	HuH-7.5	NIM811	3.11/1.19 ^3^	-	[16]
HCoV-229E-Luc	HuH-7.5	Compound 3	2.05/0.92 ^3^	-	[16]
HCoV-229E	Huh7	CsA	2.3	-	[23]
HCoV-NL63	Caco-2	CsA	2.3	-	[23]
Feline CoV	FCW	CsA	2.7	-	[23]

^1^ CPE assay, ^2^ virus yield assay, ^3^ values at 18 h/48 h post-infection.

**Table 3:** Summary of animal model studies on the effects of cyclosporines on coronavirus replication. This table summarizes in vivo studies evaluating the anti-viral effects of cyclosporines, particularly Cyclosporin A (CsA), in various coronavirus-infected animal models. It includes virus strain, animal model details, dosing regimens, routes of administration, key immunological and virological findings, and associated references.

Virus	Animal Model	Intervention	Key Findings	Reference
MERS-CoV	Mouse (Ad-hDPP4)	(CsA) administered orally at 50 mg/kg/day for 6 days.	CsA treatment significantly induced the production of mouse IFNλ (mIFNλ) in bronchoalveolar lavage fluid (BALF).	[25]
MERS-CoV	Mouse (Ad-hDPP4)	CsA at 50 mg/kg/day given orally starting 3 days post-adenoviral transduction for human DPP4 receptor expression, with MERS-CoV infection introduced intranasally on day 5 post-adenoviral infection (1.5 × 10^5^ TCID50·mL^−1^ MERS-CoV); 7 days post-infection, mice were killed, and lung tissue was used.	CsA treatment led to - significant elevation in IFNL2/3 mRNA. - decreased MERS-CoV viral load. - improved expression of the epithelial integrity marker SCNN1B. - There was also a significant reduction in lung pathology and interstitial inflammation compared to DMSO control. - Upon analysis on day seven post-infection, an inverse correlation was noted between IFNL expression and MERS-CoV levels in the lung homogenates. - The comprehensive data illustrate that the oral administration of CsA stimulates IFNλ synthesis in the pulmonary system of mice, thereby exerting significant anti-viral properties.	
SARS-CoV-2 WT	Balb/c mice expressing human ACE2 receptor.	Treatment with CsA (50 mg/kg/day) or DMSO orally for 6 days. Intranasal infection of SARS-COV-2 (1.5 × 10^4^ TCID50/mL) on day 3 after CsA treatment started. Mice were sacrificed on day 4 post-infection, and viral RNA was isolated from lung homogenate.	On day 4 post-infection, a significant decrease in SARS-CoV-2 *E* gene was detected by qPCR in mice treated with CsA compared to DMSO (*p* < 0.001).	[19]
		Treatment with CsA or DMSO (50 mg/kg/d) was initiated on the same day as infection. CsA or DMSO was applied orally for 6 days. Mice were sacrificed on day 7 post-infection, where the left lung lobe was extracted, embedded in paraffin, and stained.	Reduced infiltration of bone marrow-derived macrophages (*p* = 0.11) but unaffected recruitment of T-cells (*p* = 0.88) and neutrophils (*p* = 0.68).	

Abbreviations: MERS-CoV, Middle East respiratory syndrome coronavirus; CsA, cyclosporine A; mIFNλ, murine interferon lambda; BALF, bronchoalveolar lavage fluid; IFNL2/3 mRNA, interferon lambda 2 and 3 messenger RNA; DMSO control, dimethyl sulfoxide-treated control; IFNL, interferon lambda; SARS-CoV-2 WT, severe acute respiratory syndrome coronavirus 2 wild-type strain; TCID50, median tissue culture infectious dose; qPCR, quantitative polymerase chain reaction.

### 3.2. Clinical Trials on the Effect of Cyclophilin Inhibitors on Coronavirus Replication (Table 4)

Recent clinical investigations into SARS-CoV-2 have highlighted the potential therapeutic benefit of cyclophilin inhibitors, particularly (CsA), in combating coronavirus infections. These studies provide critical insights into efficacy, mechanisms of action, and potential limitations of cyclophilin inhibitors in the context of COVID-19 treatment. A pivotal study by [26] demonstrated that CsA administration in hospitalized COVID-19 patients *(n* = 10) resulted in significant reductions in pro-inflammatory cytokines (CXCL10, IL-10, IL-7, IL-8) and downregulation of Type 1 IFN-related gene expression. These findings corroborate the hypothesized immunomodulatory effects of cyclophilin inhibitors and their potential role in mitigating the cytokine storm associated with severe COVID-19 cases. However, the complexity of CsA’s effects was highlighted in a study by [27], which examined 20 COVID-19 patients unresponsive to dexamethasone. Despite significant increases in ferritin and WBC counts following CsA administration (10 mg/kg followed by 5 mg/kg), only 35% of patients exhibited improved lung appearance on CT scans. This discrepancy between biochemical markers and clinical outcomes underscores the need for careful interpretation of CsA’s effects and emphasizes the importance of comprehensive patient evaluation. A cohort study focusing on high-risk patients [28] reported that cyclosporine-based immunosuppression in kidney transplant recipients hospitalized with severe COVID-19 was associated with reduced mortality and respiratory complications. Notably, this regimen did not compromise renal function or induce graft rejection, suggesting a potentially favorable risk–benefit profile in this vulnerable population. The most recent and comprehensive evaluation comes from [29], who conducted a comparative study of CsA combined with standard of care (SOC) versus SOC alone. While the CsA-SOC group demonstrated a higher response rate without invasive mechanical ventilation, the difference did not reach statistical significance (*p* = 0.121). This trend, however, warrants further investigation in larger, adequately powered trials. Collectively, these clinical studies provide crucial real-world data on the effects of cyclophilin inhibitors in COVID-19 patients. They highlight the potential immunomodulatory benefits of CsA, particularly in reducing inflammatory markers and potentially improving outcomes in specific patient subgroups. However, they also underscore the variability in response and the necessity for careful patient selection and monitoring. The heterogeneity of results across these studies emphasizes the complex interplay between cyclophilin inhibition, viral replication, and host immune response. It suggests that the efficacy of cyclophilin inhibitors may be context-dependent, influenced by factors such as disease severity, timing of administration, and individual patient characteristics. Recent advancements in drug delivery systems have shown promise in enhancing the anti-viral efficacy of cyclophilin inhibitors. CsA-loaded micelles significantly outperformed CsA solution in inhibiting SARS-CoV-2 Omicron replication in Vero E6 cells [30]. The micelles formulated with TPGS demonstrated enhanced anti-viral efficacy, suggesting their potential as an intranasal delivery system for CsA in combating SARS-CoV-2 infection. Similarly, formulated CsA enhanced with 20% w/w mannitol (CsA_M20) showed superior anti-viral activity during pre-treatment and post-infection phases due to improved solubility [31]. Interestingly, different formulations of CsA have shown varying efficacies depending on the treatment strategy. While CsA_M20 was more effective in pre-treatment and post-infection scenarios, raw material CsA (CsA_rm) demonstrated higher efficacy during simultaneous treatment, possibly due to particle–virus interactions [31]. This highlights the importance of considering the timing and formulation of cyclophilin inhibitors in developing effective anti-viral strategies. The immunomodulatory effects of cyclophilin inhibitors add another dimension to their therapeutic potential. Both raw and formulated CsA significantly reduced IL-6 levels in “In THP-1/A549 co-cultures (Supplementary Table S2) [31], CsA formulations significantly reduced IL-6 levels (by ELISA), though this anti-inflammatory effect was not replicated in clinical trials (see Table 4).

**Table 4:** Summary of clinical trials on the effects of cyclosporines on coronavirus replication. This table summarizes key clinical studies evaluating the therapeutic impact of cyclosporines, particularly Cyclosporin A (CsA), in COVID-19 patients. It outlines patient populations, dosing regimens, co-administered treatments, clinical outcomes, and immunological findings. The table includes both randomized controlled trials and observational studies across different patient subgroups and clinical settings.

Virus	Population	Intervention	Key Findings	Reference
SARS-CoV-2 (COVID-19)	The study population consisted of 34 patients, with 18 in the CsA-SOC group (1 patient excluded due to ICU admission prior to trial initiation) and 16 in the SOC group. The mean age was 56.7 ± 11.8 years, 33.3% were female, and 69.7% were of Caucasian ethnicity.	CsA dosing was weight-based. At 0 h, patients received 100 mg/day for those weighing < 60 kg, 150 mg/day for those between 60 and 80 kg, and 200 mg/day for those >80 kg. At 48 h, the doses were adjusted to 150 mg/day for <60 kg, 200 mg/day for 60–80 kg, and 300 mg/day for >80 kg. The treatment duration was 1 month, with assessments conducted at 1 day, 4 days, 8 days, 30 days, and 90 days.	A higher proportion of patients in the CsA-SOC group achieved a clinical response without requiring invasive mechanical ventilation (IMV) compared to the SOC group. However, this did not reach statistical significance (*p* = 0.121).	[29]
SARS-CoV-2 (COVID-19)	A study was conducted involving 209 adult patients, with 105 assigned to the CsA group and 104 to the control group.	Oral CsA was administered at a dosage of 1–2 mg/kg/day, divided into two doses daily, for a duration of 7 days. Additionally, clarithromycin was given at 500 mg orally twice daily for 14 days, along with enoxaparin at 0.5 mg/kg/day for 14 days. Methylprednisolone was administered intravenously at 0.5 mg/kg once daily, or prednisone was given orally at 25 mg once daily, both for a period of 7 days.	Out of 149 discharged patients, 82 (55%) received CsA plus steroids, while 67 (45%) received steroids alone. Among the 60 deceased patients, 23 (38.3%) were treated with CsA plus steroids, and 37 (61.7%) received steroids alone. Data shows that patients in the CsA group had a better outcome than those with pneumonia in the progression phase.	[32]
SARS-CoV-2 WT	A total of 20 hospitalized patients (age 55.8 ± 12.9) with confirmed COVID-19 infection. Oxygen saturation ≤ 93% despite appropriate standard care for 72 h of admission, bilateral chest involvement, and unresponsiveness to dexamethasone therapy. Based in Iran. No control.	Cyclosporine (NEORAL^®^) was administered to patients who did not respond to dexamethasone therapy through saline injection over 6–8 h. Dose: 10 mg/kg followed by 5 mg/kg 24 h later.	Out of the 20 patients who received the intervention, 1. A total of 10 died. 2. A total of 10 required mechanical ventilation. 3. A total of 14 were admitted to the ICU, with a mean length of stay of 8.13 ± 6.81 days. 4. A total of 7 patients had improved lung appearance on CT scan. 5. No adverse reactions observed after cyclosporine treatment. 6. Significant (*p* < 0.05) increase in ferritin levels and WBC count after two doses of cyclosporine compared to before treatment.	[27]
SARS-CoV-2 WT	A total of 29 kidney transplant patients (median age 66.26) with PCR-confirmed SARS-CoV-2 infection. Patients were symptomatic upon admission, and all had at least one cardiovascular risk factor. 18 received supplementary oxygen during their hospital stay.	Patients were separated into two groups: 6 were randomized to minimized immunosuppressive therapy and 23 to cyclosporine-based therapy. CsA was continued in low doses in patients already on CsA. Patients on tacrolimus or mTOR inhibitors were switched to CsA. Target concentration of CsA: 50–100 ng/mL. In the minimized immunosuppressive therapy group, patients were given a reduced dose of calcineurin inhibitor.	The median CsA concentration was 60 ng/mL (IQR: 40–82.5 ng/mL). Among the patients, 3 out of 6 (50%) in the non-CsA group and 3 out of 26 (11.5%) in the CsA group died due to ARDS resulting from COVID-19. Mechanical ventilation was required for 1 out of 6 patients in the non-CsA group (16.6%) compared to 4 out of 23 (17.3%) in the CsA group. Acute kidney injury was observed in 13 patients in the CsA group upon admission, with 9 of these patients subsequently recovering. No cases of acute organ rejection or deterioration in renal function were reported in the CsA group. In non-surviving patients, inflammatory markers—such as RCP, PCT, D-dimer, ferritin, LDH, and IL-6—were significantly elevated upon admission and peaked later in the course of the disease.	[28]
SARS-CoV-2 (COVID-19)	A total of 10 hospitalized patients who required oxygen but were not in a critical condition. (median age 57.5 years)	CsA was administered at an initial dosage of 9 mg/kg/d. Median treatment duration of 4 days (range, 2–6 days), median doses received were 8 (range, 3–11 doses)	Five patients experienced adverse effects. Two discontinued treatments due to adverse events. Significant reductions (*p* ≤ 0.05) in pro-inflammatory cytokines (CXCL10, IL-10, IL-7, IL-8) were observed on day 3 post- CsA administration. Gene expression profiles in PBMCs showed downregulation of genes associated with Type 1 IFN response and innate immune cell activation post- CsA treatment.	[26]

Abbreviations: SARS-CoV-2 (COVID-19), severe acute respiratory syndrome coronavirus 2 causing coronavirus disease 2019; CsA-SOC group, cyclosporine A plus standard of care group; IMV, invasive mechanical ventilation; SARS-CoV-2 WT, severe acute respiratory syndrome coronavirus 2 wild-type strain; CT scan, computed tomography scan (imaging technique used to assess lung involvement); WBC count, white blood cell count; PCR-confirmed SARS-CoV-2 infection, infection confirmed by polymerase chain reaction testing for viral RNA; mTOR, mammalian target of rapamycin; ARDS, acute respiratory distress syndrome; RCP, reactive C-protein; PCT, procalcitonin; D-dimer, a fibrin degradation product; LDH, lactate dehydrogenase; IL-6, Interleukin 6; CXCL10, C-X-C motif chemokine ligand 10; IL-10, Interleukin 10; IL-7, Interleukin 7; IL-8, Interleukin 8; PBMCs, peripheral blood mononuclear cells; Type 1 IFN response, Type 1 interferon response.

### 3.3. Mechanism of Action of Cyclophilins and Cyclophilin Inhibitors in Coronavirus Replication (Supplementary Table S2 and Table 5)

The role of cyclophilins and (CsA) in regulating coronavirus replication involves multiple pathways, which are detailed below. Supplementary Table S2 provides a comprehensive analysis of the mechanisms described in various studies, whereas Table 5 presents a concise overview of the anti-viral actions of CsA and its derivatives for ease of reference.

**Table 5:** Summary of the mechanisms by which (CsA) and its derivatives inhibit the replication of coronaviruses. This table summarizes the different mechanisms by which CsA and its analogs exert their anti-viral activity against coronaviruses. This includes but is not limited to the inhibition of inflammatory signaling pathways, the overexpression of interferon-stimulated genes (ISGs) to adjust the host immune response, and the activation of IRF1 to amplify type I and III interferon responses. Furthermore, CsA has tissue-protective and anti-inflammatory characteristics, including the preservation of epithelial barrier function, reduction in pro-inflammatory cytokines, and maintenance of CFTR expression. Additionally, via targeting mitochondrial pathways, CsA suppresses virus-induced apoptosis and alters NFAT signaling. This facilitates the reestablishment of normal cellular functions, including directional water transport, and mitigates cytopathic consequences.

Mechanism of Action	Details	References
Modulation of host immune response	- Enhanced the expression of interferon-stimulated genes (ISGs) and interferon-beta, boosting the innate anti-viral response.	[2,25,33]
	- Suppressed inflammatory signaling pathways (STAT1, AKT, and p38) and reduced pro-inflammatory cytokines such as IL-6.	[2]
Interferon signaling and IRF1 activation	- Stimulated type I and III interferons (notably IFNλ), reducing viral load and improving lung pathology in MERS-CoV-infected models.	[25]
	- Activates IRF1-dependent anti-viral pathways, including the induction of anti-viral genes such as MX1, by promoting nuclear translocation of IRF1 independent of changes in its mRNA or protein levels.	[25,33]
	- Significant increase in IRF1 expression in response to CsA treatment.	[25]
Anti-Inflammatory and tissue-protective effects	- Decreases pro-inflammatory cytokine release and macrophage infiltration in lung tissues, reducing inflammation.	[19]
	- Significant reductions in pro-inflammatory cytokines (CXCL10, IL-10, IL-7, IL-8) observed on day 3 post-CsA administration.	[25]
	- CsA inhibits Nsp1-induced expression of IL-2 and IL-8.	[23]
	- Significantly reduced IL-6 levels by CsA	[31]
	- High concentrations of CsA significantly reduced cytokine RNA levels.	[34]
	- Maintained epithelial integrity and enhanced CFTR expression, potentially preserving lung function during infection	[25]
NFAT signaling and apoptosis modulation	- Antiviral effects are independent of both the interferon-stimulated gene response (ISRE Luciferase reporter) and the calcineurin-NFAT pathway	[35]
	- Reduced SARS-CoV Nsp1-mediated enhancement of NFAT activity, which may contribute to anti-viral effects.	[23]
	- Mitigated virus-induced apoptosis by inhibiting cyclophilin D, disrupting mitochondrial apoptotic pathways, and providing cryoprotection during coronavirus infection.	[36]
Inhibition of cytopathic effects	- CsA eliminated the cytopathic effect induced by viral infection and reduced retraction of dendrites and axons. - (CsA) and cyclophilin D (CyPD) inhibition significantly reduce viral-induced cytopathic effects (CPE) and neuronal cell death by modulating mitochondrial pathways (retaining AIF/CytC in mitochondria).	[37]
	- Protected cells from MERS-CoV-induced cytopathic effects and foci formation. - Transepithelial resistance measurements showed improved epithelial integrity in CsA-treated cells and appear to help preserve cell barrier function.	[19]
Restoring cell function	- Enhanced vectorial water transport ability in CsA-treated cells, returning to normal levels compared to infected controls.	[25]

#### 3.3.1. Role of Cyclophilins in Viral Replication

Cyclophilins, particularly cyclophilin A (CypA) and cyclophilin B (CypB), play a crucial role in coronavirus replication. Studies on feline coronavirus (FCoV) in Fcwf-4 cells demonstrated that overexpression of CypA or CypB significantly enhanced viral replication, while their knockdown or knockout substantially reduced it [38]. The peptidyl-prolyl isomerase (PPIase) domain of these cyclophilins was found to be critical for viral replication, as mutations in this domain led to reduced viral expression [38]. However, the impact of cyclophilins on viral replication appears to vary among different coronaviruses. For instance, CypA knockout in Huh7 cells showed a modest reduction in MERS-CoV replication at low MOI but had no significant effect on HCoV-229E replication [39]. Interestingly, the anti-viral effects of (CsA), a known cyclophilin inhibitor, were found to be independent of cyclophilin inhibition, suggesting a more complex mechanism of action [33]. CypA is essential for different viruses’ replication, including HCV, as demonstrated by specific knockdown experiments. Re-expression of a CypA escape mutant restored HCV replication. Mutating CypA’s hydrophobic pocket, crucial for both isomerase and chaperone activities, abolished its ability to support HCV replication. This suggests that HCV utilizes both CypA functions for efficient replication. Furthermore, the association of NS5B polymerase of HCV with CypA’s enzymatic pocket highlights a potential mechanism for CypA’s anti-viral target [40]. Conversely, in influenza A virus, CypA exhibits an anti-viral role by interacting with the M1 matrix protein. This interaction inhibits viral replication by interfering with M1’s nuclear localization. Overexpression of CypA reduces viral infectivity and promotes M1 self-association, potentially disrupting viral assembly. In contrast, CypA knockdown enhances viral replication, demonstrating its repressive effect on influenza A virus replication [41]. Together, these findings highlight the multifaceted roles of cyclophilins in viral replication, ranging from facilitators in coronaviruses and HCV to inhibitors in influenza A virus. These diverse interactions underscore the therapeutic potential of targeting cyclophilin-mediated pathways in virus-specific contexts.

#### 3.3.2. Modulation of Host Immune Response

Cyclophilin inhibitors, particularly CsA, exert their anti-viral effects partly through modulation of the host immune response. CsA treatment enhances the expression of interferon-stimulated genes (ISGs) and interferon-beta, potentially boosting the innate anti-viral response [2]. However, CsA also suppresses certain inflammatory pathways, such as STAT1, AKT, and p38 signaling, and reduces pro-inflammatory cytokine production, including IL-6 [2,31]. (CsA) modulates macrophage polarization during influenza A virus infection in mice, reducing pro-inflammatory (M1) and enhancing anti-inflammatory (M2) phenotypes. This effect, dependent on CsA binding to (CypA), is mediated through regulation of IFN-γ/STAT1 and IL-4/STAT6 signaling pathways. By shifting macrophage polarization, CsA attenuates inflammatory responses, revealing a novel mechanism for its immunomodulatory role in viral infections [42]. This dual action of immune modulation may contribute to its anti-viral efficacy while potentially mitigating excessive inflammation.

#### 3.3.3. Interferon Signaling and IRF1 Activation

A key mechanism of CsA’s anti-viral action involves the stimulation of type I and III interferons, particularly IFNλ. In MERS-CoV-infected cells and animal models, CsA significantly induced IFNλ production, correlating with reduced viral load and improved lung pathology [25]. The induction of anti-viral genes, such as MX1, by CsA was found to be IRF1-dependent, with IRF1 knockout significantly reducing CsA’s anti-viral efficacy [33]. CsA treatment did not alter IRF1 mRNA or protein levels in HCoV-229E-infected A549 cells. However, CsA exposure for 48 h induced a significant nuclear translocation of IRF1, suggesting a potential regulatory role of CsA on IRF1 activity independent of its transcriptional regulation [25,33]

#### 3.3.4. Anti-Inflammatory and Tissue-Protective Effects

Beyond its direct anti-viral actions, CsA exhibits anti-inflammatory properties that may be beneficial in managing coronavirus infections. In human precision-cut lung slices (hPCLs), CsA treatment reduced pro-inflammatory cytokine release and decreased macrophage infiltration [19]. Additionally, CsA enhances epithelial integrity and CFTR expression, potentially preserving lung function during infection [25].

#### 3.3.5. NFAT Signaling and Apoptosis Modulation

While CsA is known to inhibit the calcineurin-NFAT pathway, studies suggest that its anti-viral effects against coronaviruses are largely independent of this mechanism [35]. However, CsA does interfere with the SARS-CoV Nsp1-mediated enhancement of NFAT activity, potentially contributing to its overall anti-viral effect [23]. CsA also demonstrates anti-apoptotic properties in the context of coronavirus infection. In SADS-CoV-infected cells, CsA significantly reduced virus-induced apoptosis, possibly through inhibition of cyclophilin D and subsequent disruption of mitochondrial apoptotic pathways [36]. This cytoprotective effect may contribute to CsA’s overall efficacy in managing coronavirus infections.

### 3.4. Limitations

- Off-Target Effects: CsA, in particular, has well-documented immunosuppressive effects, which can increase the risk of infections and other adverse events [25,31]. This property has limited its direct application as an anti-viral therapy.
- Limited Clinical Data: While preclinical studies have shown promising results, there is a need for more extensive clinical trials to evaluate the efficacy and safety of these compounds in human patients.
- Inconsistent Reporting of Antiviral Potency: Among the in vitro studies on cyclophilin inhibitors, some reported EC50 values, others included both EC50 and CC50, while several did not report cytotoxicity at all. This inconsistency makes cross-comparison of results challenging and may lead to differences in perceived efficacy and safety across studies [2,10,13,14,17].
- Nephrotoxicity and Metabolic Side Effects: Cyclosporine A is associated with dose-dependent nephrotoxicity, hypertension, and metabolic disturbances, which pose significant challenges for long-term clinical use. These adverse effects necessitate careful therapeutic drug monitoring and patient management, thereby limiting its utility as a broadly applicable anti-viral agent [43].

### 3.5. Future Directions

- Novel Cyclophilin Inhibitors: Since CsA and some of its derivatives cause immunosuppression, developing non-immunosuppressive cyclophilin inhibitors with retained anti-viral potency is a promising strategy to improve safety and broaden clinical use.
- Combination Therapy: Exploring combination therapies with other anti-viral agents or immunomodulatory drugs may enhance the therapeutic efficacy of cyclophilin inhibitors.
- Targeted Drug Delivery: Developing targeted drug delivery systems to deliver cyclophilin inhibitors specifically to infected cells could improve their efficacy and reduce side effects.
- Mechanistic Studies: Further research is needed to elucidate the precise mechanisms of action of cyclophilin inhibitors and their interactions with host factors and viral proteins.
- Clinical Trials: Large-scale clinical trials are necessary to evaluate the safety and efficacy of cyclophilin inhibitors in treating COVID-19 and other coronavirus infections.

## 4. Conclusions

Cyclophilin inhibitors, particularly (CsA) and its derivatives, demonstrate significant potential as broad-spectrum anti-viral agents against coronaviruses. These compounds exhibit multifaceted mechanisms of action, including direct inhibition of viral replication, modulation of host immune responses, and cytoprotective effects. The efficacy of cyclophilin inhibitors across various coronavirus strains and their ability to synergize with other anti-viral agents highlights their promise as therapeutic interventions for current and future coronavirus outbreaks.

## Data Availability

Not applicable.

## Supplementary Materials

The following supporting information can be downloaded at https://www.mdpi.com/article/10.3390/ijms26167900/s1. References [22,23,24,31,37,44,45] were cited in Supplementary Materials.
