# Supplementary material for: Broad-Spectrum Antiviral Activity of Cyclophilin Inhibitors Against Coronaviruses: A Systematic Review
Source: Int J Mol Sci. 2025 Aug 15;26(16):7900. doi: 10.3390/ijms26167900 (PMC12386573; doi:10.3390/ijms26167900)
Supplement: Supplementary file 1 [file ijms-26-07900-s001.zip › ijms-3762516-supplementary.pdf]

Note: all citations in the supplementary file can be found in the main manuscript.

Virus	Cells	Intervention details: drug, dose, and duration	Outcome	Other comments/findings	Reference
HCoV-229E	phBECs	CsA and ALV, 10 µM, with and without pretreatment	Significant inhibition of intra- and extracellular viral replication.	Inhibition is more pronounced in pretreated cells. Non-toxic concentrations used.	[17]
HCoV-229E -RLuc	Huh-7.5	CsA and ALV, 10,20, and 30 µM, post-infection only	Drastic decrease in Renilla luciferase activity. 2 log reduction in RLU at 10 µM ALV and 4 log reduction at 10 µM CsA.	Minimal dose-dependent cytotoxicity, more pronounced for CsA.	
SARS-CoV-1 Replicon	HEK293	CsA and ALV, 10,20, and 30 µM, post-infection only	Drastic decrease in Renilla luciferase activity. 2.5 log reduction in RLU at 10 µM ALV and 3.5 log reduction at 10 10 µM CsA.	Moderate, dose-dependent cytotoxicity for CsA; negligible for ALV.	
HCoV-229E-Luc	HuH-7.5	CsA at 0,5,10,15, and 20 µM for 18 and 48 hours post-infection	EC50 2.09 and 0.97 µM at 18 and 48 hours, respectively.		[16]
		ALV at 0,5,10,15, and 20 µM for 18 and 48 hours post-infection	EC50 2.77 and 1.37 µM at 18 and 48 hours, respectively		
		NIM811 at 0,5,10,15, and 20 µM for 18 and 48 hours post-infection	EC50 3.11 and 1.19µM at 18 and 48 hours, respectively		

		compound 3 at 0,5,10,15, and 20 μM for 18 and 48 hours post-infection	EC50 2.05 and 0.92 μM at 18 and 48 hours, respectively		
		CsA, ALV, NIM-811, and compound **3 at** 0–20 μM, 48 hours.	Decreased N protein expression between 1.25 and 5 μM on western blot analysis.		
MERS-CoV (ECM)	Vero cells	9 μM cyclosporine A; 2.4 × 10^4 U/ml IFN-α1; both separately and combined. Duration: 24, 48, and 72 hours	Significant reduction in viral titres with both individual and combined treatments. Combined treatment was most effective.		[2]
	Ex vivo human bronchus culture	9 μM cyclosporine A; 2.4 × 10^4 U/ml IFN-α1; both separately and combined. Evaluated at 1,8,24,40, and 56 hours post-infection	In bronchus cultures, combined treatment significantly reduced viral titres compared to single treatments.	The additive or synergistic effects of cyclosporine A and IFN-α1 are evident in limiting virus replication more effectively than either treatment alone in bronchus cultures.	
	Ex vivo human lung culture	9 μM cyclosporine A; 2.4 × 10^4 U/ml IFN-α1; both separately and combined. Evaluated at 1,8,24,40, and 56 hours post-infection	Combination therapy or cyclosporine A alone significantly reduced viral titres compared to untreated or IFN-α1 treatment alone. The effect of cyclosporine A alone was similar to the combination therapy.	Cyclosporine A, both alone and in combination with IFN-α1, effectively reduces viral replication in lung tissues, suggesting a potent antiviral effect.	
	HMVEC-L	9 μM cyclosporine A; 2.4 × 10^4 U/ml IFN-α1; both separately and combined. Evaluated at 1,24, and 48 hours post-infection	The group treated with a combination of IFN-α1 and CsA exhibited the most substantial decrease in HCoV-EMC replication using both the TCID-50 assay and NP protein expression(western blot).		

SARS-CoV-GFP	Vero E6 or 293/ACE2	Treatment with CsA ranged from 0 to 64 µM post-infection for 1 hour; cells were fixed at 18 hours p.i.	CsA reduced GFP expression in a dose-dependent manner, with significant inhibition at 16 µM for Vero E6 cells and similar results in 293/ACE2 cells.	CsA demonstrated efficacy in halting SARS-CoV-GFP replication in both monkey and human cell lines without impacting cell health. Low doses of CsA (≤4 µM) may slightly enhance SARS-CoV replication.	[18]
HCoV-229E-GFP	Huh-7	Treatment with CsA ranged from 0 to 64 µM post-infection for 1 hour; cells were fixed at 24 h p.i.	Complete inhibition of GFP expression required 32 µM CsA.	HCoV-229E-GFP was less sensitive to CsA compared to SARS-CoV-GFP and MHV-GFP(complete inhibition at 16 µM in 17CL1 cells), with cell viability unaffected by CsA.	
SARS-CoV–GFP	Vero E6	CsA treatment from 1 to 10 h p.i., doses ranging from 0 to 32 µM	Significant reduction in SARS-CoV nsp8, N protein, and GFP expression at 16 µM CsA.		
wt SARS-CoV		CsA treatment from 1 to 10 h p.i. with a concentration of 16 µM	Nearly undetectable levels of nsp8 and N protein expression with 16 µM CsA.	The replication of both recombinant and wild-type SARS-CoV was similarly inhibited by CsA, with a marked effect observed between 8 and 16 µM CsA.	
SARS-CoV		CsA treatment from 1 to 10 h p.i., concentrations from 0 to 32 µM	Immunofluorescence analysis showed reduced nsp4 and dsRNA levels with increasing CsA concentration, especially at 16 µM.		
SARS-CoV–GFP / wt SARS-CoV		Treatment with various CsA concentrations from 1 h p.i., virus titers measured at 16 h p.i.	Progeny virus titers significantly reduced by 16 µM CsA, showing a 3-4 log decrease.	CsA significantly hindered the production of infectious progeny for SARS-CoV, aligning with the suppression of viral protein and RNA synthesis.	

HCoV-229E–GFP	Huh-7	CsA treatment from 1 h p.i. onwards, at 0,16 and 32 µM. virus titers measured at 30 h p.i.	2-log reduction in virus titers with 32 µM CsA.	MHV progeny titer was reduced by 2 logs upon 16 µM treatment in 17CL1 cells.	
MERS-CoV	Vero	CsA treatment at 3 or 9 µM for 3 days post-infection.	CsA at 9 µM prevented CPE, maintaining cell viability comparable to mock-infected controls.	CsA showed potential as an antiviral by preserving cell health and inhibiting viral CPE in Vero cells.	[21]
	Huh7	CsA treatment at 3.75, 7.5, or 15 µM	Treatment reduced or completely prevented CPE at 7.5 and 15 µM, respectively.	CsA effectively inhibited CPE in Huh7 cells, confirming its antiviral effect against MERS-CoV.	
SARS-CoV-2(Omicron BA.1)	Vero E6	Protocol A: Treatment 1 hour before infection	Protocol A: -<br>Both HL and ML micelles showed a maximum antiviral activity of about 70%.<br>LL micelles showed a similar effect to blank micelles.	-CsA micelles(TPGS) for nasal delivery<br>HL micelles(0.5 mg/mL),<br>ML micelles(0.25 mg/mL),<br>LL micelles(LL: 0.1 mg/mL),<br>-Raw CSA<br>Blank formulations at concentrations (32,16,8,4,2µM)	[30]
		Protocol B: Treatment at the time of infection	Protocol B: -<br>increased antiviral activity with increasing CSA concentrations, leading to values above 90% of viral inhibition for HL, ML, and LL at 32, 8, and 4 µM, respectively.<br>LL micelles show 107% antiviral activity at 8 µM.<br>Raw CSA is Less effective, with 100% antiviral efficiency only at 32 µM.		

			Blank micelles show 7-79% antiviral activity, less effective than drug-loaded micelles.		
		Protocol C: Post-treatment 2 hours after infection	Protocol C: -<br>HL micelles exhibited a 102% viral inhibition, while the ML micelles exhibited a 111% viral inhibition. The LL micelles' effectiveness was proportional to the CSA concentration, with a peak in correspondence with the 8 μM drug concentration (101%).<br>Raw CSA shows 45% antiviral activity at 32 μM, less effective at lower doses.<br>Blank micelles show<br>38-66% antiviral activity, less effective than drug-loaded micelles.		
		Protocol C6: Post-treatment 6 hours after infection	Protocol C6: -<br>The lowest percentages of viral inhibition for all three different developed micellar formulations were obtained (77% at 32 μM, 73% at 16 μM, 73% at 8 μM for HL, ML, and LL micelles, respectively).<br>Raw CSA shows 42% antiviral activity at 32 μM, less effective at lower doses<br>Blank micelles show <40% antiviral activity, less effective than drug-loaded micelles.		
		Protocol D: Pre-treatment 1 hour before infection, followed by post-treatment 2 hours after infection	Protocol D: -<br>The HL and ML micelles showed a similar trend consisting of a very high antiviral activity, never going under		

			90%, with the highest values at 2 μM (122% for HL and 135% for ML micelles).<br>LL micelles were slightly different, *i.e.,* consisted of a peak of activity (116%) at 8 μM but lower values at decreasing CSA concentrations, however, never going under 100% inhibition.<br>Raw CSA shows 39% antiviral activity at 32 μM, ineffective at lower doses<br>Blank micelles show <74% antiviral activity, less effective than drug-loaded micelles.		
		Protocol E: Three post-treatments spaced 1 hour apart	Protocol E: -<br>The HL and ML micelles showed high antiviral activity in all the conditions tested (never under 70%), with the highest values recorded at 2 μM (109% for HL and 106% for ML micelles).<br>LL micelles show 106% antiviral activity at 8 μM<br>Raw CSA shows <28% antiviral activity<br>Blank micelles show 88% antiviral activity, less effective than drug-loaded micelles.		
		Protocol F: Pre-treatment 1 hour before infection, followed by three post-treatments spaced 1 hour apart	Protocol F: -<br>HL and ML micelles were again very high (always above 90%), with the highest value (116% for the HL and 121% for the ML micelles) recorded at 2 μM CSA concentration.<br>The LL micelles' peak of antiviral activity (115%) was observed at the	Overall, our findings revealed that micelles encapsulating CSA (esp. ML and HL) demonstrated a superior antiviral performance when compared to the simple CSA solution and micelles without the drug, as assessed by qRT-PCR for N-gene.	

			highest CSA concentration tested (8 µM).<br>Raw CSA shows <25% antiviral activity<br><br>Blank micelles show 84% antiviral activity, less effective than drug-loaded micelles		
HCoV-229E	A549	CsA, conc. .1 to 10 µM, applied 2 h.p.i for 48 hours. MOI 0.01. DMSO was used as a control.	CsA effectively suppressed HCoV-229E infection in a dose-dependent manner, achieving an IC50 of 0.15 µM, while not impacting cell viability in the given conditions. (viral titre was measured in the supernatant).	CsA reduced the HCoV-229E N gene in a dose-dependent manner. Reduction in the expression of the inhibitory effect was stable at MOI 0.01 or 0.1. Moreover, a parallel decrease in N protein expression was detected via Western blot analysis at a CsA concentration of 10 µM.	[33]
MERS-CoV	Calu-3 cells and Primary human alveolar epithelial cells (hAECs)	10 µM CsA for 24 hpi	- Reduced MERS-CoV RNA levels by >95%(quantitative PCR).<br>- 2.6 to 2.8 log_{10} reduction in the release of infectious MERS-CoV particles ($TCID_{50}$).<br>- Reduced formation of cell foci and apoptosis		[25]

SARS-CoV-2	Calu-3	Voclosporin(VCS) or CsA or Tacrolimus(TAC) at concentrations from 0 to 100 µM for 24 hours. Tissue culture was done in glassware to increase the bioavailability of the lipophilic compounds.	Administering 3.2 µM of VCS (in its pure form) to the cells resulted in a reduction of SARS-CoV-2 infectious progeny titers by over 1.5 logs. In contrast, applying the same dosage of CsA or TAC led to a reduction of approximately 0.5 logs.	The virus concentration in the culture medium from infected Calu-3 cells was quantified via plaque assay on Vero E6 cells using supernatants harvested 24 hours post-infection. Treatment with 3.2 µM of VCS or CsA, however, induced cytotoxicity, reducing cell viability to approximately 75%.	[44]
Ec50	Vero	Alisporivir 0-50 µM all are incubated for 3 days, except MERS-CoV EMC/2012 in HuH7 for 2 days.	EC50 was calculated to be 3.6 ± 1.1 µM by CPE assay and 3.9 ± 1.7 µM by virus yield assay.	Cell toxicity observed at higher ALV concentrations, with half-maximal cytotoxic concentration (CC50) at 26.4 µM.	[20]
MERS-CoV(EMC/2012)	Huh7		EC50 was calculated to be 3.4 ± 1.0 µM by CPE assay and 2.8 ± 1.0 µM by virus yield assay.	ALV toxicity was low, with a CC50 value of 43.8 µM.	
MERS-CoV(EMC/2012)	LLC-MK2		EC50 was calculated to be 4.0 ± 1.1 µM by virus yield assay.	ALV toxicity was low, with a CC50 value of 14.3 ± 1.8.	
MERS-CoV (N3/Jordan strain)	Vero		ALV showed efficacy against this strain as well, with an EC50 of 3.0 ± 1.0 µM by CPE-based assay.	ALV toxicity was low, with a CC50 value of 26.4 ± 1.0	
MERS-CoV (N3/Jordan strain)	HuH7		EC50 of 1.5 ± 1.0µM by CPE-based assay.	Cell viability was well-maintained with a CC50 of 43.8 µM.	

SARS-CoV(Frankfurt-1)	VeroE6		EC50 of 8.3 ± 1.0μM by CPE-based assay.	Cell viability was well-maintained with a CC50 of >50 μM.	
SARS-CoV(MA-15)	VeroE6		EC50 of 1.3 ± 0.05 μM by CPE-based assay.	Cell viability was well-maintained with a CC50 of >50 μM.	
MERS-CoV	Vero	ALV, 3.1-6.3 μM, starting 1 hr p.i., assessed at 48 hr p.i.	Approximately 2 log reduction in virus titer at 6.3 μM assessed by plaque assay of virus yield.		
	Huh7	ALV, 3.1-12.5 μM, starting 1 hr p.i., assessed at 48 hr p.i.	Approximately 2 log reduction in virus titer at 3.1 μM assessed by plaque assay of virus yield.		
	HAE cells from two donors	ALV, 25 μM, starting 1 hr p.i., assessed at 48 hr p.i.	Approximately 3 log reduction in virus titer at 25 μM assessed by plaque assay of virus yield in HAE cells from donor#2.		
SARS-CoV	Vero	ALV, 3.1-6.3 μM, starting 1 hr p.i., assessed at 32 hr p.i.	Approximately 5 log reduction in virus titer at 6.3 μM assessed by plaque assay of virus yield.		
	VeroE6	ALV, 6.3-25 μM, starting 1 hr p.i., assessed at 32 hr p.i.	Approximately 1.5 log reduction in virus titer at 6.3 μM assessed by plaque assay of virus yield.		
MHV	17Cl1	ALV, 6.3-25 μM, starting 1 hr p.i., assessed at 16 hr p.i.	Approximately 1-log reduction in progeny titers at 6.3 μM ALV, and a 2-log reduction at 25 μM.		
HCoV-229E	Huh7	ALV, 6.3-12.5 μM, starting 1 hr p.i., assessed at 16 hr p.i. and 48 hr p.i.	~1.5 log reduction in virus titer at a concentration of 6.3 μM and a ~2 log reduction at 12.5 μM, as assessed by plaque assay.		

[redacted]

SARS-COV-2	CaLu3	CsA,10 µM pre & post SARS-COV-2 infection at an MOI 0.05. Samples were analysed 48hpi.	Western blot analysis revealed that CsA-treated cells, both pre- and post-infection, exhibited a 95% reduction in SARS-CoV-2 spike (S) protein expression compared to DMSO-treated controls. Similarly, CsA treatment resulted in approximately a 98% reduction in N1 protein expression, as assessed by droplet digital PCR (ddPCR). Furthermore, pre-treatment with CsA led to a ~98% decrease in intracellular viral particles (IVP), while post-infection treatment demonstrated a 90% reduction in IVP levels.	Cell viability was neither affected by viral infection nor drug treatment (MTT assay)	[34]
SARS-COV-2(B.1.1.7)	CaLu3	CsA 0.1,1, 10 µM pre or post-infection with 0.05 MOI SARS-COV-2. Samples analysed 48 hpi.	A dose-dependent reduction in viral RNA levels was observed across all CsA concentrations. A strong correlation was identified between viral RNA levels and intracellular viral particles (IVP) in the supernatants of CsA-treated cells infected with the B.1.1.7 variant, suggesting that this variant predominantly releases infectious virions.	CsA is effective even at concentrations compatible with clinical practice.	
[redacted]					
SARS-CoV-2 WT	HBEpCs (human bronchial	MOI 0.1. Treatment with 10µM CSA or DMSO (control) for 24h. Without pre-treatment. Viral gene	At 24h post-infection, a significant reduction in SARS-CoV-2 *E* gene expression (p-value = 0.027) and viral	No cytotoxic concentrations used.	[19]

	epithelial cells)	expression detected by qPCR and viral titre determined by TCID50.	titre (p-value = 0.031) in CSA-treated cells compared to DMSO.		
	hPCLSs (human precision-cut lung slices)	Infected lung slices treated with 10μM CSA or DMSO for 72h. Viral gene expression detected by qPCR and viral titre determined by plaque assay.	At 72h post-infection, a significant reduction in SARS-CoV-2 *E* gene expression (p-value = 0.02) and viral titre (p-value = 0.0009) in CSA-treated hPCLs compared to DMSO.	hPCLs treated with CsA showed recovery in metabolic activity and decreased release of pro-inflammatory cytokines (TNF-alpha, p=0.136; IL-1beta, p=0.22; IFN-gamma, p=0.013).	
	HBEpCs	Cells infected with SARS-CoV-2 variants α (B.1.1.7), β (B.1.351), or zeta (P.2) at MOI 0.1. Infected cells were treated with 10μM CSA or DMSO for 24 hours.	CsA inhibited replication of all three variants compared to DMSO.		
HCoV-NL63 WT	Caco-2	MOI 0.004 and treated with increasing concentrations of CSA or non-immunosuppressive CSA derivatives (ALV, NIM811, compound 3), as well as FK508 (Tacrolimus).	1.For CSA, EC50=0.9-2.0μM.<br>2. For ALV, EC50=0.8μM.<br>3. For NIM811, EC50=0.8μM.<br>4. For compound 3, EC50=1.1 μM.<br>5.For FK508, EC50=6.6 μM.	No cytotoxic concentrations used.	[24]
		Infected cells were treated with either CSA, ALV, or NIM811 at 0, 1.25, 5, and 20μM.	Significant decrease in the detected N-protein at concentrations between 1.25 and 5.00μM of all drugs tested(western blot)	Drugs inhibit the replication cycle of the virus.	
SARS-COV-2	Vero E6	Cells were infected for 2h at an MOI of 0.02 in the presence of increasing	Alisporivir reduced SARS-CoV-2 RNA production in a dose-dependent manner (RT-qPCR)	For comparison EC50 of chloroquine was 0.35 μM.<br>No cytotoxic concentrations used	[22]

		conc. of Alisprovir or chloroquine (+ve control). After 48h incubation virus was quantified by RT-qPCR	EC 50 was 0.46 µM, EC90 was 3.10 µM. the maximum viral was 2 log at 5 µM.		
SARS-COV-2	Vero E6	Cells were infected at an MOI of 0.4 in the presence of increasing conc. of Alisporivir; after virus removal, infected cells were incubated for 24 hours in the presence of Alisporivir and assessed by immunofluorescence using anti-dsRNA antibody	Alisporivir reduced the number of SARS-CoV-2-infected cells in a dose-dependent manner and attained complete inhibition at 10 µM.		
SARS-COV-2	Vero E6	Cells were infected at an MOI 0.4 for 2h in the presence of 5µM of Alisporivir or chloroquine. After virus removal, the cells were incubated for 7 hours in the absence of the compounds; fixed, and immunostained with anti-dsRNA antibody.	No infected cells were detected with chloroquine at 5 µM, proving that it prevents viral entry to the cell. in contrast, Alisporivir did not inhibit virus entry into the cell. Antiviral effects of Alv were preserved when added 3h pi, while completely abolished when added 6h pi.	Results suggest that Alisporivir inhibits post-entry steps SARS-COV-2 lifecycle.	
FIPV	*Felis catus* whole fetus-4 (FCWF-4)	cells have been infected with MOI of 1(pfu), after adsorption of 1hr at 37°c. Medium containing virus was removed, and cells were rinsed with PBS and incubated with various	CsA inhibits intracellular FIPV replication in vitro, but not by FK506<br><br>CsA treatment reduced the levels of FIPV N protein in a dose-dependent manner (western blot), whereas FK506	CsA's antiviral effects against FIPV in fcwf-4 cells are independent of both the interferon-stimulated gene response (ISRE Luciferase reporter) and the calcineurin-NF-AT pathway (NF-AT luciferase reporter), suggesting involvement of other mechanisms.	[35]

		conc. of CsA (0-6.3 µM) and FK506 (0.08-10 µM)for 20hr. Giemsa staining was done for plaque counting.	did not significantly impact FIPV N protein levels.<br><br>Quantitative RT-PCR shows suppression of FIPV RNA replication by CsA, whereas FK506 shows inhibitory effect only at 10µM		
SADS-CoV	Vero E6	Cells were pre-treated with CsA (10 µM)or DMSO for 1hr then infected and dual labelled with annexin and PI, SADS-COV-induced apoptosis quantified by Annexin V/PI flow cytometry at 12h, 24h, and 48h.	Treatment with CsA notably decreases the percentage of SADS-COV-induced apoptotic cells during infection		[36]
SADS-CoV	Vero E6	Cells were pre-incubated with CsA (10µM) or Z-VAD-FMK (100 µM) for 1h, then infected with SADS-CoV.	DNA laddering assay indicated that Z-VAD-FMK or CsA treatment in SADS-CoV-infected cells completely abolished intracellular DNA fragmentation (Agarose gel electrophoresis).	CsA-mediated CypD inhibition effectively suppressed SADS-CoV-induced apoptosis, indicating that SADS-CoV triggers the mitochondrial apoptotic pathway.	
SADS-CoV	Vero E6	Cells were pretreated with CsA 5 and 10 µM or DMSO for 1h, then infected with SADS-CoV. Analyzed 36hpi	CsA had a strong inhibitory effect on SADS-CoV proliferation by significantly reducing N protein expression. (Inverted fluorescence microscope.)<br><br>The number of cells expressing viral antigen, as quantified by N protein staining, decreased during CsA treatment, with almost 100% inhibition observed at the 10 µM concentration		

SADS-CoV	Vero E6 and IPI-2I	Cells were pretreated with CsA 5 and 10 µM or DMSO for 1h, then infected with SADS-CoV for 36h. For immunostaining, cells were fixed 36hpi	CsA (10 µM) significantly prevented the intracellular expression of the viral N protein (WB). Pretreatment with the inhibitor significantly decreased SADS-CoV-induced cleavage of PARP. (WB)	CsA treatment significantly reduced the release of viral progeny in a dose-dependent manner (Spearman-Karber method).	
[redacted]					
SARS-CoV (Frankfurt-1)	Vero E6	MOI 0.0001. Virus replication measured by real-time RT-PCR and plaque titration. Post-treatment with differing concentrations of CSA.	EC50 of 3.3µM.	No significant cytopathic effects. CSA inhibits genome replication as opposed to viral entry.	[23]
HCoV-229E	Huh-7	MOI 0.1	EC50 of 2.3µM.		
HCov-NL63	CaCo-2	MOI 0.004	EC50 of 2.3µM.		
Feline CoV	FCW	MOI (NOT MENTIONED)	EC50 of 2.7 µM		

Supplementary Table S1 | Summary of cell culture studies on the effects of cyclosporines on coronavirus replication

This table summarizes experimental conditions used in cell culture studies assessing the effects of cyclosporines and non-immunosuppressive cyclophilin inhibitors on coronavirus replication. It includes cell types, effective concentrations (EC_{50}), and key findings.

Abbreviations: HCoV-229E, Human coronavirus 229E; CsA, Cyclosporine A; ALV, Alisporivir; µM, Micromolar; HCoV-229E-RLuc, Human coronavirus 229E expressing Renilla luciferase; RLU, in Renilla luciferase activity; SARS-CoV-1 Replicon, Severe acute respiratory syndrome coronavirus 1 replicon; HCoV-229E-Luc, Human coronavirus 229E expressing luciferase; EC50, Half maximal effective concentration; NIM811, Nonimmunosuppressive cyclosporine analogue; MERS-CoV (ECM), Middle East respiratory syndrome coronavirus; IFN-α1, Interferon alpha 1; HCoV-EMC, Human coronavirus Erasmus Medical Center strain; TCID-50 assay, Median tissue culture infectious dose assay; NP protein expression, Nucleocapsid protein expression; SARS-CoV-GFP, Severe acute respiratory syndrome coronavirus expressing green fluorescent protein; p.i, Post-infection; GFP expression, Green fluorescent protein expression; HCoV-229E-GFP, Human coronavirus 229E expressing green fluorescent protein; MHV-GFP, Murine hepatitis virus expressing green fluorescent protein; SARS-CoV nsp8, Non-structural protein 8 of severe acute respiratory syndrome coronavirus; wt SARS-CoV, Wild-type severe acute respiratory syndrome coronavirus; nsp4, Non-structural protein 4 of coronavirus; MHV progeny titer, Quantity of infectious Murine Hepatitis Virus particles produced; CPE, Cytopathic effect (visible cell damage caused by viral infection); SARS-CoV-2 (Omicron BA.1), Severe acute respiratory syndrome coronavirus 2, Omicron variant BA.1;

HL and ML micelles, High-loading and medium-loading micelles (drug delivery systems); LL micelles, Low-loading micelles (drug delivery systems); TPGS, D-alpha-tocopheryl polyethylene glycol succinate (a solubilizing agent and drug delivery enhancer); qRT-PCR, Quantitative reverse transcription polymerase chain reaction; N-gene, Nucleocapsid gene; conc, Concentration; h.p.i., Hours post-infection; IC50, Half-maximal inhibitory concentration; MOI 0.01 or 0.1, Multiplicity of infection 0.01 or 0.1; VCS, Voclosporin; TAC, Tacrolimus; MERS-CoV (EMC/2012), Middle East respiratory syndrome coronavirus Erasmus Medical Center/2012 strain; CC50, 50% cytotoxic concentration; MERS-CoV (N3/Jordan), Middle East respiratory syndrome coronavirus N3/Jordan strain; SARS-CoV (Frankfurt-1), Severe acute respiratory syndrome coronavirus Frankfurt-1 strain; SARS-CoV (MA-15), Severe acute respiratory syndrome coronavirus MA-15 strain; DMSO-treated controls, Dimethyl sulfoxide-treated control groups; ddPCR, Droplet digital polymerase chain reaction; IVP, intracellular viral particles; MTT assay, 3-(4,5-dimethylthiazol-2-yl)-2,5-diphenyltetrazolium bromide assay; SARS-CoV-2 (B.1.1.7), Severe acute respiratory syndrome coronavirus 2 Alpha variant (lineage B.1.1.7); MOI SARS-CoV-2, Multiplicity of infection for SARS-CoV-2; SARS-CoV-2 WT, Severe acute respiratory syndrome coronavirus 2 wild-type strain; TNF-alpha, Tumor necrosis factor alpha; IL-1beta, Interleukin 1 beta; IFN-gamma, Interferon gamma; HCoV-NL63 WT, Human coronavirus NL63 wild-type strain; FK506, Tacrolimus; RT-qPCR, Reverse transcription quantitative polymerase chain reaction; EC90, Effective concentration for 90% maximal response; anti-dsRNA antibody, Antibody targeting double-stranded RNA; FIPV, Feline infectious peritonitis Virus; pfu, Plaque-forming units; PBS, Phosphate-buffered saline; ISRE Luciferase reporter, Interferon-stimulated response element luciferase reporter; calcineurin-NF-AT pathway, Calcineurin-nuclear factor of activated T-cells pathway; SADS-CoV, Swine acute diarrhea syndrome coronavirus; Annexin V/PI, Annexin V and propidium iodide staining (used to detect apoptotic and necrotic cells); Z-VAD-FMK, Pan-caspase inhibitor; WB, Western blot; PARP, Poly (ADP-ribose) polymerase; SARS-CoV (Frankfurt-1), Severe acute respiratory syndrome coronavirus Frankfurt-1 strain; HCoV-NL63, Human coronavirus NL63; Feline CoV, Feline coronavirus.

Virus	Cells	Intervention details: drug, dose, and duration	Outcome	Other comments/findings	Reference
MERS-CoV (ECM)	Ex vivo human lung and bronchus culuture	Combined treatment with 9 µM CsA and 2.4 × 10^4 U/ml IFN-α1. Evaluated at 24 hours post-infection (hpi) for lung tissue and 56 hpi for bronchial tissue.	Induction of interferon-stimulated genes (ISGs) in treated tissues, with significant gene expression increases compared to untreated tissues. SGs such as ISG15, MX1, IRF7, IFI44, IFI44L, OAS1, SP110, IFIT1, IFIT2, IFIT3, and IFI27 were highly induced as revealed by qPCR	The combination of CsA and IFN-α1 did not increase expression of interferon receptors (IFNAR1, IFNAR2, and IFN gamma receptors) but did enhance expression of specific ISGs and IFN beta-1.	[2]
	HMVEC-L	IFN-α1 at 2.4 × 10^4 U/ml and/or CsA at 9 µM, applied to cultures for 1 hour at 37°C.	CsA led to a significant decrease in STAT1, AKT, and p38 activation, while it had no effect on m-Tor activation.	Jak-STAT and AKT/mTOR pathways are probably not involved in the induction of ISGs upon combined CsA and IFN-α1 treatment.	

			-Combined treatment did not activate STAT1, AKT , mTOR and p38 compared with IFN-α1 treatment .		
HCoV-229E	A549,	MOI 0.01. CsA or DMSO 10 µM, 48 hours	Increased MX1(ISG) expression measured by RT-qPCR of cell lysate.	CsA treatment enhances ISG expression in multiple cell lines, regardless of their tumorigenic status or origin.	[33]
	BEAS-2B	MOI 0.5. CsA or DMSO 10 µM, 48 hours	Increased MX1(ISG) expression measured by RT-qPCR of cell lysate.		
	Huh7	MOI 0.01. CsA or DMSO 10 µM, 48 hours	Increased MX1(ISG) expression measured by RT-qPCR of cell lysate.		
	A549	CsA or DMSO 10 µM, RT-qPCR of MX1 at 6,12,24,48, and 72 hpi. Cells were infected at an MOI of 0.01.	CsA triggers the expression of MX1 at 48- and 72-hours post-infection (h.p.i), with no significant induction at earlier intervals.		
	A549 (CypA KO, CypB KO)	CsA or DMSO 10 µM, 48 hours, MOI of 0.01. Cyclophilin A and B knockout cell lines were created with CRISPR/Cas 9.	CsA triggers the expression of MX1 at 48 hpi.	The induction of antiviral gene expression MX1 by CsA does not depend on the presence of CypA or CypB, as demonstrated by experiments using knockout cell lines.<br>Western blot confirmed successful knockout of cyclophilin A and B.	
	A549 CypA KO, CypB KO	CsA or DMSO 10 µM, 48 hours, MOI 0.01. Cyclophilin A and B knockout cell lines were created with CRISPR/Cas 9.	CsA significantly reduces N gene expression irrespective of CypA or CypB presence. N gene expression by RT-qPCR at 48 hpi.		

HCoV-229E N and SARS-CoV-2 N	293T/17	Co-transfection with N- and empty vector control reporters, treatment with 10 µM CsA or DMSO for 24 hours, and poly(I: C) stimulation.	The cells were simultaneously transfected with either the 229E N or SARS-CoV-2 N coding plasmids, or with a control plasmid along with a plasmid carrying an IFN-β firefly luciferase reporter gene.	CoV N proteins suppressed IFN-β activation; CsA did not mitigate suppression.	
HCoV-229E	A549	Mock or HCoV-229E infection MOI 0.01, with 10 µM CsA or DMSO, for 48h. poly(I:C) used as an activator positive control for IRF3.	IRF3 luciferase reporter activity was measured.	HCoV-229E infection reduced IRF3 activity; CsA did not reverse the suppression of IRF3 activity.	
		Mock or HCoV-229E infection (MOI 0.01), treated with 10 µM CsA or DMSO for 48h. TNF-α serves as a positive control for NF-κB activation.	NF-κB reporter activity (SEAP activity measured in the supernatant).	HCoV-229E infection did not notably alter NF-κB activity; similarly, CsA treatment did not impact NF-κB activity, with	
		Uninfected and infected (MOI 0.01) cells were treated with 10 µM CsA or DMSO for 48 h.	CsA increased MX1 expression (RT-qPCR) in uninfected cells , but to a lesser extent compared to infected cells.		
HCoV-229E	A549	CsA 10 µM or DMSO, with or without 5 µM Rux for 48 h. MOI 0.01.	229E N expression and MX1 expression were assessed by qPCR. Viral titer was also determined by plaque assay.	CsA induction of MX1 expression and suppression of viral replication were not impacted by Rux, indicating CsA acts independently of JAK/STAT signaling. Ruxolitinib (Rux) is an inihibitor of type I	

				and III interferon signalling (Jak-STAT pathway). The intervention with 5 μM ruxolitinib impeded IFN-β's inhibitory action on HCoV-229E infection.	
		CsA or DMSO 10 μM for 48 h.	CsA selectively induced expression of certain ISGs(MX1, OAS2, RSAD2) but not TLR3 or ISG15 by RT-qPCR.	This suggested a targeted modulation of antiviral pathways rather than a broad induction of the IFN response.	
HCoV-229E	A549 IRF1 KO	CsA 10 μM or DMSO for 48 h. Cells infected (MOI 0.01) or uninfected.	MX1 induction by CsA is significantly diminished in the absence of IRF1 (in uninfected cells)	IRF1 knockout confirmed by Western blot. CsA's impact on MX1 expression is IRF1-dependent.	
			MX1 expression induction in HCoV-229E-infected cells was absent in IRF1 knockout cells.	IRF1's role in CsA-mediated MX1 expression was confirmed.	
			10-fold decrease in CsA's antiviral effect in infected IRF1 KO cells (plaque assay on supernatant).	The antiviral potency of CsA against HCoV-229E is partially mediated by IRF1.	
HCoV-229E	A549	CsA 10 μM or DMSO for up to 48 h. MOI 0.01.	No significant change in IRF1 expression upon CsA treatment at 6, 12, 24, or 48hpi.	IRF1 expression levels were quantified by RT-qPCR and confirmed through Western blot, with no significant changes observed with CsA treatment.	
		CsA 10 μM or DMSO for 48 h.	Significant increase in nuclear localization of IRF1 with CsA treatment for 48 hours. This effect was not observed upon CsA treatment for 6 or 24 hours.	Cellular distribution of IRF1 shifted towards the nucleus following CsA treatment, as shown by immunofluorescence microscopy and quantified through image analysis. CsA may modulate IRF1 activity through altering its subcellular localisation.	

MERS-CoV	Calu-3 cells	10 µM CsA for 24 hpi	-Protected cells from MERS-CoV-induced cytopathic effects and foci formation.<br><br>-Inhibited MERS-CoV-induced apoptosis (Caspase-3/7 Glo® Assay).<br>-Improved expression of CFTR protein and ENaCβ after CsA treatment in infected cells (western blot).	Suggests a protective effect of CsA on cells.	[25]
	Calu-3 cells grown on transwell filters	10 µM CsA for 48 hpi	Transepithelial resistance measurements showed improved epithelial integrity in CsA-treated cells compared to infected controls.	CsA appears to help preserve cell barrier function.	
			Fluorescein isothiocyanate-dextran quantification showed enhanced vectorial water transport ability in CsA-treated cells, returning to normal levels compared to infected controls.	Suggests CsA aids in restoring cell function.	
MERS-CoV	Calu-3 cells and hAECs	-CsA (10 µM, 24 hours)<br>-ALV (10 µM, 24 hours)<br>-CnA inhibitor (20 µM, 24 hours)<br>-NFAT inhibitor (50 µM, 24 hours)	Both CsA and ALV significantly decreased virus titers, whereas CnA and NFAT inhibitors did not lead to significant changes. JNK inhibitor reduced virus titers, but not p38 inhibitor. CsA and ALV also significantly	-The effect of all treatments on viral replication was assessed in both cell lines using TCID-50 determination and viral RNA.<br>-The suppressive influence on viral replication by CsA and ALV is independent	

		-JNK inhibitor (SP600125, 10 µM, 24 hours)<br>-p38 MAPK inhibitor (SB 203 580, 10 µM, 24 hours)	lowered viral RNA levels, unlike the other inhibitors which did not reduce viral RNA including JNK inhibitor.	of CnA, NFAT, JNK, or p38 pathway inhibition.	
	Calu-3 cells	CsA (10 µM, 24 hours)	CsA stimulation led to a pronounced upregulation of genes related to antiviral responses and interferon signaling, including significant increases in IFNB1(Type I IFN) and IFNL1/2(Type II IFN) expression. Many interferon-stimulated genes (ISGs) were also elevated.	CsA treatment may prompt an antiviral state in lung epithelial cells.	
		Cyclosporin A (CsA): 10 µM, times post-treatment for analysis ranged from 15 to 56 hours	-Confirmation of elevated expression in various ISGs like MxA, PKR, OAS1, IFIT1, IFIT2, IFIT3, Bst2/tetherin, RSAD2/viperin, and XAF1 was also observed at 18 hours post-treatment.<br>-Elevation in IFNB mRNA (up to a 57-fold increase).<br>- IFNL1 and IFNL2/3 mRNA showed significant upsurges (150 to 387-fold).<br>-Protein levels of IFNλ1 and IFNλ3 were robustly induced by CsA as early as 12 hours post-treatment, with peak values of 4222±890 ng/mL at 48 to 56 hours post-treatment in the supernatant. (ELISA)	The enhancement of IFN-related responses indicates a distinctive antiviral state induced by CsA in pulmonary epithelial cells, with the quantification of IFNλ proteins supported by ELISA.	
		CsA: 10 µM, 4 hours post-treatment	IRF1 mRNA levels were markedly increased with CsA treatment, while	Upregulation of IRF1 suggests its specific role in IFNL gene expression.	

			IRF3, IRF7, and IRF9 were not significantly altered(qPCR)		
		Enhanced expression of IRF1 in CsA-stimulated cells was observed via immunofluorescence.	Enhanced expression of IRF1 in CsA-stimulated cells was observed via immunofluorescence.	IRF1 is a potential mediator of IFNλ expression induced by CsA.	
		CsA: 10 µM, 3 and 4 hours post-treatment.	Significant increase in IRF1-expressing cells in response to CsA treatment by immunofluorescence quantification.	The quantification underscores the specific induction of IRF1 by CsA.	
		CsA: 10 µM, siRNA treatments	Knockdown of IRF1 with siRNA led to a significant decrease in IFNL mRNA levels in CsA-treated cells(qPCR).	Implicates IRF1 in the regulation of IFNL 1 and 2/3 expression following CsA treatment.	
		CsA: 10 µM, siRNA treatments	siRNA-mediated silencing of IRF1 resulted in over 75% inhibition of IFNλ protein release in CsA-treated cells(ELISA) compared to control.	Demonstrates the critical role of IRF1 in IFNλ protein production after CsA stimulation.	
MERS-CoV	Calu-3	IRF1 siRNA transfection; CsA: 10 µM, 24h post-infection	IRF1 knockdown led to a significant rise in viral particles released from CsA-treated cells (TCID-50)	Demonstrates the pivotal role of IRF1 in mediating the antiviral effects of CsA.	
		Neutralising antibodies for IFNλ1/2/3 or IFNβ; CsA treatment	Anti-IFNλ1/2/3 antibodies increased viral titres by 1.05 log_{10} level compared to control in the presence of CsA, while anti-IFNβ did not.	Suggests that IFNλ, rather than IFNβ, is involved in the CsA-induced reduction in MERS-CoV replication.<br>These data indicate that the antiviral effects of CsA were at least partially	

				mediated by an IRF1–IFNλ signalling axis, and independent of type I IFN.	
[redacted]					
SARS-COV-2 strains (EU, B.1.1.7 and P .1)	Calu3 cells	CsA 0.1,1, 10 µM pre or post-infection with 0.05 MOI SARS-COV-2. Samples analysed 48 hpi.	RT-PCR analysis revealed that high concentrations of CsA (1 µM and 10 µM) significantly reduced cytokine RNA levels in both experimental conditions. In contrast, CsA at 0.1 µM was ineffective in reducing cytokine RNA expression.	The lowest concentration of CsA (0.1 µM) was found to be more effective against the EU strain of SARS-CoV-2 compared to other variants. For the EU strain, CsA exhibited a dose-dependent effect. These findings collectively support the anti-inflammatory action of CsA in infected lung cells.	[34]
SARS-COV-2	Calu3 cells	CyPA expression was silenced using shRNA transduction, and after 12 days, the cells were infected with SARS-CoV-2 at a multiplicity of infection (MOI) of 0.05. Samples were collected and analyzed 48 hours post-infection (hpi).	-Western blot analysis revealed an increase in S protein levels in CyPA-silenced cells.<br>-Droplet digital PCR (ddPCR) analysis showed that N1 protein levels were elevated in CyPA-silenced cells compared to non-targeting (NT) shRNA-transduced controls.<br>- Increase in intracellular viral particles (IVP) and higher viral titers were observed in the supernatants of CyPA-silenced cells, as assessed by TCID50.<br>-CyPA knockdown did not significantly affect CD147 but showed ACE2 expression changes. (Immunostaining and Western blotting.).<br>-Changes in cytokine levels were noted in CyPA-silenced cells. (RT-PCR)	CyPA acts as a negative modulator of SARS-CoV-2, suggesting that the inhibitory effects of CsA on the virus likely involve a different molecular mechanism.	
[redacted]					

	THP-1/A549 co-cultures	CsA_rm at 10 μg/mL, CsA_M20 at 10 μg/mL of CsA, mannitol (Mann) at 2 μg/mL, followed by LPS for 24 hours.	-Significantly reduced IL-6 levels by CsA (both raw and formulated compared to the vehicle.<br><br>-Mannitol also showed a slight anti-inflammatory effect, but not statistically significant.<br><br>-The inhaled CsA powder maintained its anti-inflammatory properties and could limit the cytokine storm(ELISA).	CsA and mannitol were prepared in two distinct ratios: 80:20 (CsA_M20). CsA_rm refers to raw CsA without the addition of mannitol.<br><br>Viability of A549 and THP-1 cell cultures was not affected by the various CsA-treated treatments.	[31]
SARS-CoV-2 Omicron BA.1	Vero E6	CsA_rm or CsA_M20 was administered at concentrations of 9.6, 19.2, 38.4, and 76.9 μg/mL in relation to three treatment protocols:<br><br>-Pre-treatment: Administered one hour before infection.<br>-Post-treatment: Administered two hours after infection.<br>-Simultaneous treatment: Administered during infection."	-In the pre-treatment protocol, CsA_rm and CsA_M20 at a concentration of 76.9 μg/mL resulted in a reduction of viral infectivity by 58% and 78%, respectively; however, no significant effects were observed at lower concentrations.<br><br>-In the post-infection protocol, both CsA_rm and CsA_M20 demonstrated a reduction in viral infectivity across all tested concentrations, except 9.6 μg/mL. Notably, CsA_M20 exhibited greater efficacy than CsA_rm in this context.<br><br>During simultaneous treatment, both CsA_rm and CsA_M20 effectively reduced viral infectivity at all concentrations. Interestingly, CsA_rm	CsA_M20 demonstrates greater efficacy than CsA_rm under pre-treatment and post-infection conditions, likely due to the enhanced solubility and dissolution of the composite particles.<br><br>Conversely, CsA_rm exhibits superior effectiveness compared to CsA_M20 during simultaneous infection. This may be attributed to an interaction between the CsA_rm suspension and the virus, where the insoluble particles interact with the viral cell membrane.	

			exhibited a 30% greater antiviral effect compared to CsA_M20 at concentrations of 19.2 and 9.6 µg/mL."		
[redacted]					
HCoV-NL63 WT	Caco-2	WT CaCo-2 cells and CaCo-2 cells in which cyclophilin A or B were knocked down through the use of lentiviral shRNA expression vectors. Control cell line transduced with lentiviral non-target control gene. Plaque titration assay was used to determine the growth of HCoV-NL63 in WT and mutant CaCo-2 cells.	CypA knockdown (ΔCypA) resulted in a ~4 log reduction in virus titer, while CypB knockdown (ΔCypB) had no significant effect on virus replication.	Western blot and qPCR confirmed that cyclophilin A and B genes were successfully knocked down in CaCo-2 cells.	[24]
[redacted]					
FCoV	Fcwf-4	Felis catus whole fetus-4 (Fcwf-4) cells were transfected with pEF-Myc-CypA, pEF-Myc-CypB, or the pEF/Myc-His empty vector as a control. At 24 hours post-transfection, the cells were selected using blasticidin, and individual resistant colonies were cloned. These cells were infected with FCoV.	Western blot analysis shows increased viral replication in transfected cells. While RT-qPCR results reveal a 1.8-fold increase in the FCoV-N gene expression in cells stably expressing CypA, and a 3.2-fold increase in those expressing CypB.		[38]
	Fcwf-4	Knockdown (KD) of CypA using shRNAs.	CypA knockdown resulted in about an 80% decrease in CypA expression and a		

			corresponding 65-80% reduction in viral copies and progeny.		
	Fcwf-4	Knockdown (KD) of CypB using shRNAs.	KD of CypB reduced viral protein expression and viral copies by approximately 70%		
	Cyp A KD Fcwf-4	Transfection with pEF-Myc-CypA.	Reintroducing CypA restored viral replication to levels similar to control cells		
	Cyp B KD Fcwf-4	Transfection with pEF-Myc-CypA	Reintroducing CypA restored viral replication to levels similar to control cells		
	fcwf-4 cells	CRISPR/Cas9 knockout of CypA	>95% reduction in FCoV replication		
	fcwf-4 cells	CRISPR/Cas9 knockout of CypB	>95% reduction in FCoV replication		
	fcwf-4 cells	Mutations in PPIase domain (Transfection with CypA mutants (R55A, F60A, H126Q))	Reduced FCoV-N expression and viral copies	Mutation of PPIase domain causes decreased isomerase activity, leading to decreased viral replication.	
	fcwf-4 cells	Mutations in the PPIase domain (Transfection with CypB mutants (R62A, F67A)	Reduced FCoV-N expression and viral copies		
EAV	Huh7-CypAKO, pool	CypA knockout (pool)	~2-log reduction in virus titers compared to control cells	Indicates partial dependence on CypA for EAV replication; pool has variable knockout levels	[39]

	Huh7-CypAKO clones #1 and #2	CypA knockout (clone)	~3-log reduction in virus titers compared to control cells	Stronger dependence on CypA for EAV replication; clonal lines show complete knockout	
	Huh7-CypBKO, CypCKO, CypDKO, pool	CypB, CypC, CypD knockout (pool)	No significant change in virus yield compared to control cells	EAV replication is not dependent on CypB, CypC, or CypD in pooled cells	
MERS-CoV	Huh7-CypAKO, pool	CypA knockout (pool)	No significant change in virus yield compared to control cells	Pool knockout may still express low levels of CypA, sufficient for MERS-CoV replication	
	Huh7-CypAKO clones #1 and #2	CypA knockout (clone)	~3-fold reduction in virus replication at low MOI (0.01); no effect at high MOI (5)	Clones show more complete knockout of CypA, affecting replication at low MOI only	
	Huh7-CypBKO, CypCKO, CypDKO, pool	CypB, CypC, CypD knockout (pool)	No significant change in virus yield compared to control cells	MERS-CoV replication is not affected by the knockout of CypB, CypC, or CypD	

HCoV-229E	Huh7-CypAKO, pool	CypA knockout (pool)	No significant change in virus yield compared to control cells	Low levels of residual CypA in pooled cells are likely sufficient for HCoV-229E replication	
	Huh7-CypAKO clones #1 and #2	CypA knockout (clone)	No effect on virus yield compared to control cells	HCoV-229E replication is not dependent on CypA, even in clonal knockout lines	
	Huh7-CypBKO, CypCKO, CypDKO, pool	CypB, CypC, CypD knockout (pool)	No effect on virus yield compared to control cells	HCoV-229E replication is not affected by the knockout of CypB, CypC, or CypD	
SARS-CoV	HEK293	Cyclophilins and SARS-CoV Nsp1 were cloned into LUMIER assay vectors and transfected into HEK293 cells. Calcineurin/NFAT pathway stimulated by the addition of PMA and ionomycin.	Expression of Nsp1 does not induce NFAT activity directly but increases the stimulation of PMA/ionomycin on NFAT. The increase in NFAT activity is blocked by CSA.		[23]
	HEK293	HEK293 cells co-transfected with interleukin reporter plasmids (IL-2, IL-4, IL-8 luc)	• Nsp1 significantly induced expression of IL-2. However, this effect is inhibited by CSA.		

		and expression plasmids encoding NFAT3, calcineurin, and SARS-CoV Nsp1 full length.	• Nsp1 did not induce IL-4 expression significantly.<br>• IL-8 expression was significantly increased in the presence of PMA/ionomycin but significantly reduced in the presence of Nsp1 and completely inhibited with CSA.		
HCoV-OC43 WT (rOC/ATCC)	LA-N-5 (neuronal cell line)	MOI 0.2 and incubated for 2h. Infected cells were treated with 2.5µM CSA for 24, 48, and 72 hours post-infection.	CsA eliminated the cytopathic effect induced by viral infection and reduced retraction of dendrites and axons.		[37]
		LA-N-5 (Cyclophilin D knocked down using shRNA). 2 knockdown populations, M and K, were created. MOI 0.2 and incubated for 2h.	Cyclophilin D knocked eliminated the cytopathic effect induced by viral infection and reduced retraction of dendrites and axons.	Population K only expressed 6.75% of the cyclophilin D expressed in normal cells. Population M expressed 34.55%. Greater reduction in cytopathic effects in population K compared to population M. Viability of infected population K is similar to mock-infected cells.<br>Viability of infected population M is similar to mock-infected cells at 24 and 48h, but significant reduction at 72h.	
HCoV-OC43 (rOC/U(S 183-241))	LA-N-5	MOI 0.2 and incubated for 2h. Infected cells were treated with 2.5µM CSA for 24, 48, and 72 hours post-infection.	CsA induced a moderate reduction in cytopathic effects, less than that observed for HCoV-OC34 WT.	At 48h post-infection, slight improvement in neuronal survival in cells treated with CSA.	
		LA-N-5 (Cyclophilin D knocked down using shRNA). 2 knockdown populations, M and K, were created. MOI 0.2	Cyclophilin D knockdown induced a moderate reduction in cytopathic effects, less than that observed for HCoV-OC34 WT.		

		and incubated for 2h. MOI 0.2 and incubated for 2h.			
HCoV-OC43 WT (rOC/ATCC) and (rOC/U(S 183-241))	LA-N-5	Caspase activity of infected human neuron was quantified by using LEHD-PNa for caspase-9 AND DEVD-pNa for caspase-3<br><br>Cells were treated with the virus and treated with either the pan caspase inhibitor (VAD) or caspase-9 inhibitor (LEHD) for 24,48,72h pi	Both caspase-9 and caspase-3 were activated in human neurons at 48h pi by at least twofold.<br><br>Effective inhibition of Indicator caspase-9 and effector caspase-3 did not impair or delay neuronal death, which clearly indicates that neuronal PCD induced by both viruses is not inhibited by Z-VAD-FMK and strongly suggests that caspases are not essential factors in the process.	rOc/u s183 produced stronger activity as compared to rook/ATCC.<br><br>The presence of both inhibitors did not affect the cell viability (MTS-PMS assay).	
HCoV-OC43 WT (rOC/ATCC) and (rOC/U(S 183-241))	LA-N-5	Cells were infected and treated with CsA, and the detection of AIF by immunofluorescence	Inhibition of CyP D impairs the nuclear translocation of AIF (in infected neurons), which affects virus-induced cell death.<br><br>WB shows that CyPD inhibition slightly favoured the retention of AIF and CytC in mitochondria following infection.		
[redacted]					
SARS-COV	Bacterial cells M-15(protein expression)	Mapping interactions between host proteins and SARS-NP, using SPR (surface plasma resonance).<br><br>Binding responses were tested for SARS_NP injected at conc.	NP of SARS-COV binds hCypA with high affinity (KD) ranging from 6-160nm, which is 100 times higher than HIV-1 CA to hCypA.		[45]

		31.25,62.5,125,250,500 and 1000 Nm.	SARS_NP–hCypA interaction is 100–2700 times stronger than HIV-1 CA–hCypA interaction. This finding suggests a possible target for anti-SARS therapies.		
		Analysis of SARS-NP-hCypA binding has been performed.<br><br>Site-directed mutagenesis	The binding model predicted SARS_NP loop Trp302-Pro310 interacts with hCypA. Mutagenesis confirmed crucial residues in both proteins.<br>- SARS_NP Mutations: Trp302Ala reduced affinity (disrupted cation–π interaction), Ile305Ala reduced affinity (interacts with hCypA Asn102), reduced affinity, Gln307Ala reduced affinity (disrupted hydrogen bonds).<br>- hCypA Mutations: Arg55Ala reduced affinity (disrupted hydrogen bonds with Gln307), Gln63Ala reduced affinity (disrupted interactions with Ile305), Trp121Phe reduced affinity (disrupted hydrogen bond with Phe308).<br>- Energetic Analysis: SPR and MM/PBSA methods showed consistency, validating the model. The unexpected increase in binding affinity when Arg69 was		

			mutated to Ala was explained by reduced water molecules at the interface.		

Supplementary Table S2 | Mechanistic insights into cyclosporine and cyclophilin activity in coronavirus infections

This table compiles key mechanistic studies exploring the molecular actions of cyclosporines and cyclophilins across various coronaviruses. It includes in vitro, ex vivo, and genetic knockout studies, highlighting drug dosages, cell types, targeted pathways, and downstream effects on viral replication, immune signaling (e.g., IFNs, ISGs, IRF1), and host-virus interactions. It also outlines the functional roles of specific cyclophilin isoforms (e.g., CypA, CypB, CypD).

Abbreviations: MERS-CoV (ECM), Middle East respiratory syndrome coronavirus Erasmus Medical Center strain; µM, Micromolar; CsA, Cyclosporine A; IFN-α1, Interferon alpha 1; hpi, Hours post-infection; IS, Interferon-stimulated; Gs, Guanine nucleotide-binding proteins (G proteins); ISG15, Interferon-stimulated genes including ISG15; MX1, Myxovirus resistance protein 1; IRF7, Interferon regulatory factor 7; IFI44, Interferon-induced protein 44; IFI44L, Interferon-induced protein 44-like; OAS1, 2'-5'-Oligoadenylate synthetase 1; SP110, Speckled 110 kDa protein; IFIT1, Interferon-induced protein with tetratricopeptide repeats 1; IFIT2, Interferon-induced protein with tetratricopeptide repeats 2; IFIT3, Interferon-induced protein with tetratricopeptide repeats 3; IFI27, Interferon alpha-inducible protein 27; qPCR, Quantitative polymerase chain reaction; IFNAR1, Interferon alpha/beta receptor 1; IFNAR2, Interferon alpha/beta receptor 2; IFN gamma, Interferon gamma; IFN beta-1, Interferon beta-1; STAT1, Signal transducer and activator of transcription 1; AKT, Protein kinase B; p38 activation, Activation of p38 mitogen-activated protein kinase; m-Tor activation, Activation of mammalian target of rapamycin; Jak-STAT, Janus kinase-signal transducer and activator of transcription pathway; AKT/mTOR pathways, Signaling pathways involving AKT and mammalian target of rapamycin; HCoV-229E, Human coronavirus 229E; MOI 0.01, Multiplicity of infection of 0.01; DMSO, Dimethyl sulfoxide; MX1 (ISG) expression, Expression of MX1 interferon-stimulated gene; RT-qPCR, Reverse transcription quantitative polymerase chain reaction; CRISPR/Cas9, Clustered regularly interspaced short palindromic repeats/CRISPR-associated protein 9; CypA, Cyclophilin A; CypB, Cyclophilin B; HCoV-229E N and SARS-CoV-2 N, Nucleocapsid proteins of human coronavirus 229E and SARS-CoV-2; poly(I:C) stimulation, Stimulation with polyinosinic:polycytidylic acid; the 229E N, Nucleocapsid protein of HCoV-229E; SARS2 N coding plasmids, Plasmids encoding the nucleocapsid protein of SARS-CoV-2; IRF3, Interferon regulatory factor 3; TNF-α, Tumor necrosis factor alpha; NF-κB, Nuclear factor kappa-light-chain-enhancer of activated B cells; SEAP activity, Secreted embryonic alkaline phosphatase activity; 229E N expression, Expression of nucleocapsid protein from human coronavirus 229E; Rux, Ruxolitinib; IRF1, Interferon regulatory factor 1; Caspase-3/7 Glo® Assay, A luminescent assay measuring caspase-3 and caspase-7 activity; CFTR protein, Cystic fibrosis transmembrane conductance regulator protein; ENaCβ, Epithelial sodium channel beta subunit; ALV, Alisporivir; CnA inhibitor, Calcineurin A inhibitor; NFAT inhibitor, Nuclear factor of activated T-cells inhibitor; JNK inhibitor, c-Jun N-terminal kinase inhibitor; MAPK inhibitor, Mitogen-activated protein kinase inhibitor; p38 inhibitor, p38 mitogen-activated protein kinase inhibitor; TCID-50, Median tissue culture infectious dose; MxA, Myxovirus resistance protein A; PKR, Protein kinase R; OAS1, 2'-5'-Oligoadenylate synthetase 1; IFIT1, Interferon-induced protein with tetratricopeptide repeats 1; IFIT2, Interferon-induced protein with tetratricopeptide repeats 2; IFIT3, Interferon-induced protein with tetratricopeptide repeats 3; Bst2/tetherin, Bone marrow stromal antigen 2 (also known as tetherin, an antiviral protein); RSAD2/viperin, Radical S-adenosyl methionine domain-containing 2; XAF1, XIAP-associated factor 1; ELISA, Enzyme-linked immunosorbent assay; IRF7, Interferon regulatory factor 7; IRF9, Interferon regulatory factor 9; siRNA treatments, Small interfering RNA treatments; IFNL mRNA levels, Interferon lambda messenger RNA levels; SARS-CoV-2 strains (EU, B.1.1.7, and P.1), Variants of severe acute respiratory syndrome coronavirus 2 including European lineage, Alpha variant (B.1.1.7), and Gamma variant (P.1); CyPA expression, Cyclophilin A expression; shRNA transduction, Short hairpin RNA-mediated gene silencing via viral vector transduction; ddPCR, Droplet digital polymerase chain reaction; CD147, Cluster of differentiation 147; ACE2, Angiotensin-converting enzyme 2; CsA_rm, raw Cyclosporine without the addition of mannitol; CsA_M20, Cyclosporine with addition of mannitol; Mann, Mannose; LPS, Lipopolysaccharide; SARS-CoV-2 Omicron BA.1, Omicron variant BA.1 of SARS-CoV-2; HCoV-NL63 WT, Human coronavirus NL63 wild-type strain; FCoV, Feline coronavirus; pEF-Myc-CypA, Expression plasmid encoding Myc-tagged Cyclophilin A; pEF-Myc-CypB, Expression plasmid encoding Myc-tagged Cyclophilin B; pEF/Myc-His, Expression plasmid vector with Myc and His tags for protein expression; KD, Knockdown; PPIase domain, Peptidyl-prolyl cis-trans isomerase domain; CypA mutants (R55A, F60A, H126Q), Mutated forms of Cyclophilin A with specific amino acid substitutions at residues 55, 60, and 126; CypB mutants (R62A, F67A), Mutated forms of Cyclophilin B with specific amino acid substitutions at residues 62 and 67; EAV, Equine arteritis virus; CypC, Cyclophilin C; CypD, Cyclophilin D; Nsp1 cloned, Non-structural protein 1 cloned; LUMIER assay vectors, Plasmid vectors used for LUMIER (LUminescence-based Mammalian IntERactome); PMA, Phorbol 12-myristate 13-acetate; luc, Luciferase; NFAT3, Nuclear factor of activated T-cells 3 ; HCoV-OC43 WT (rOC/ATCC), Human coronavirus OC43 wild-type strain from ATCC; mock-infected cells, Cells treated identically but without virus infection (control group); HCoV-

OC43 (rOC/U S183-241), Recombinant HCoV-OC43 with spike protein mutations at positions 183 and 241; LEHD-pNa, A synthetic peptide substrate used to measure caspase-9 activity; DEVD-pNa, A synthetic peptide substrate used to measure caspase-3 activity; LEHD, Caspase-9 recognition sequence; neuronal PCD, Neuronal programmed cell death; rOC/U S183, Recombinant OC43 virus with mutation at spike position 183; rOC/ATCC, Recombinant OC43 virus from ATCC strain; MTS-PMS assay, A cell viability assay using MTS tetrazolium compound and PMS electron coupling reagent; AIF, Apoptosis-inducing factor; SARS-NP, SARS coronavirus nucleocapsid protein; SPR, Surface plasmon resonance; hCypA, Human cyclophilin A; SARS_NP–hCypA interaction, Interaction between SARS nucleocapsid protein and human cyclophilin A; SARS_NP loop Trp302-Pro310, Loop region of SARS nucleocapsid protein from tryptophan 302 to proline 310; Trp302Ala, Tryptophan to alanine mutation at position 302; disrupted cation–π interaction, Loss of cation–pi non-covalent interaction; Ile305Ala, Isoleucine to alanine mutation at position 305; hCypA Asn102, Asparagine at position 102 in human cyclophilin A; Gln307Ala, Glutamine to alanine mutation at position 307; Arg55Ala, Arginine to alanine mutation at position 55; Trp121Phe, Tryptophan to phenylalanine mutation at position 121; Phe308, Phenylalanine at position 308; MM/PBSA, Molecular mechanics Poisson–Boltzmann surface area; Arg69, Arginine at position 69; Ala, Alanine.
